# Dynamic transmission modeling of COVID-19 to support decision-making in Brazil: A scoping review in the pre-vaccine era

**DOI:** 10.1371/journal.pgph.0002679

**Published:** 2023-12-13

**Authors:** Gabriel Berg de Almeida, Lorena Mendes Simon, Ângela Maria Bagattini, Michelle Quarti Machado da Rosa, Marcelo Eduardo Borges, José Alexandre Felizola Diniz Filho, Ricardo de Souza Kuchenbecker, Roberto André Kraenkel, Cláudia Pio Ferreira, Suzi Alves Camey, Carlos Magno Castelo Branco Fortaleza, Cristiana Maria Toscano

**Affiliations:** 1 Department of Infectious Diseases, Dermatology, Imaging Diagnosis, and Radiotherapy, Botucatu Medical School (FMB), São Paulo State University (Unesp), Botucatu, São Paulo State, Brazil; 2 Department of Ecology, Postgraduate Programme in Ecology and Evolution, Federal University of Goiás (UFG), Goiânia, Goiás State, Brazil; 3 Institute of Tropical Pathology and Public Health, Federal University of Goiás (UFG), Goiânia, Goiás State, Brazil; 4 Observatório Covid-19 BR, São Paulo, São Paulo State, Brazil; 5 Postgraduate Programme of Epidemiology, Federal University of Rio Grande do Sul (UFRGS), Porto Alegre, Rio Grande do Sul State, Brazil; 6 Institute for Theoretical Physics, São Paulo State University (Unesp), São Paulo, São Paulo State, Brazil; 7 Department of Biodiversity and Biostatistics, Institute of Biosciences (IBB), São Paulo State University (Unesp), Botucatu, São Paulo State, Brazil; 8 Department of Statistics, Institute of Mathematics and Statistics, Federal University of Rio Grande do Sul (UFRGS), Porto Alegre, Rio Grande do Sul State, Brazil; Fundacao Oswaldo Cruz, BRAZIL

## Abstract

Brazil was one of the countries most affected during the first year of the COVID-19 pandemic, in a pre-vaccine era, and mathematical and statistical models were used in decision-making and public policies to mitigate and suppress SARS-CoV-2 dispersion. In this article, we intend to overview the modeling for COVID-19 in Brazil, focusing on the first 18 months of the pandemic. We conducted a scoping review and searched for studies on infectious disease modeling methods in peer-reviewed journals and gray literature, published between January 01, 2020, and June 2, 2021, reporting real-world or scenario-based COVID-19 modeling for Brazil. We included 81 studies, most corresponding to published articles produced in Brazilian institutions. The models were dynamic and deterministic in the majority. The predominant model type was compartmental, but other models were also found. The main modeling objectives were to analyze epidemiological scenarios (testing interventions’ effectiveness) and to project short and long-term predictions, while few articles performed economic impact analysis. Estimations of the R_0_ and transmission rates or projections regarding the course of the epidemic figured as major, especially at the beginning of the crisis. However, several other outputs were forecasted, such as the isolation/quarantine effect on transmission, hospital facilities required, secondary cases caused by infected children, and the economic effects of the pandemic. This study reveals numerous articles with shared objectives and similar methods and data sources. We observed a deficiency in addressing social inequities in the Brazilian context within the utilized models, which may also be expected in several low- and middle-income countries with significant social disparities. We conclude that the models were of great relevance in the pandemic scenario of COVID-19. Nevertheless, efforts could be better planned and executed with improved institutional organization, dialogue among research groups, increased interaction between modelers and epidemiologists, and establishment of a sustainable cooperation network.

## Introduction

Mathematical and statistical modeling are essential tools for studying infectious diseases [1, 2]. From significant problems of nosocomial infection and transmission of pathogens between patients or even hospitals, passing through evaluation of the introduction of new vaccines for centuries-old diseases, to predictions and describing the dynamic transmission of new epidemics and pandemics, such as the HIV emergency, those models have proven it selves valuable [3–6].

Models are considered a scientific and transparent way to analyze increasingly complex issues and are well applied when data gaps are identified. Since the emergence of SARS-CoV-2 in early 2020, international entities and governments have developed and used various models in public response [7]. Short- and long-term predictions, analysis of epidemiological scenarios, and evaluation of non-pharmacological intervention measures, for example, were subjects assessed [8].

In addition, mathematical and statistical models were essential to understanding the principles of SARS-CoV-2 transmission dynamics and numerically estimating fundamental conceptions of the new virus. Data on the first 425 cases in Wuhan, China, were analyzed to determine epidemiologic characteristics of the novel coronavirus pneumonia [9]. The incubation period was estimated by fitting a log-normal distribution, while the serial interval was estimated by fitting a gamma distribution. A transmission model derived the epidemic growth rate and doubling time. Using a different methodology, combining a stochastic model with real data on cases of COVID-19 between December 2019 and February 2020 from Wuhan and outside Wuhan, for example, Kucharski et al. simulated how transmission from human to human varied over time and estimated the basic reproductive number [10]. Many other authors, using various methodologies, estimated the essential parameters [11–13].

Furthermore, straight at the beginning of the novel coronavirus pandemic, the Imperial College of London, for example, developed compartmental-based modeling capable of predicting, from publicly available data, the evolution of the number of cases, hospitalizations, and deaths from the USA and U.K. [14]. This fact was crucial as it made the U.K. government rethink strategies against SARS-CoV-2. Before the publication of the models, the government was planning a herd immunity strategy. Forewarned of the overload of the health system and the predicted excess of deaths, a change to mitigation and viral suppression strategies was carried out [15].

International organizations aiming to expand the reach of mathematical and statistical modeling and provide information to decision-makers have spearheaded global initiatives. For example, the COVID-19 International Modeling Consortium (CoMo Consortium) brought together infectious disease modelers and public health experts from over 40 countries to develop an age-structured compartmental model to estimate the trajectory of COVID-19 based on different scenarios [16]. A user-friendly platform with a web-based interface has been created, allowing researchers and policymakers to utilize the tool for real-time impact prediction. Another noteworthy initiative is the CMCC (COVID-19 Multi-Model Comparison Collaboration), a group composed of governments, foundations, and development partners that support actions for the COVID-19 response, primarily in low- and middle-income countries (LMICs). The group aimed to study and provide information regarding several models for COVID-19, their main objectives, capabilities, and limitations [17].

However, as modeling needs to be developed for a specific scenario, we must incorporate many parameters to mimic reality. The availability and quality of the data can directly affect the results. Decision-making in public policies also may affect the results. For example, mass testing public policies or choosing to test only severe cases can also introduce bias when calibrating the parameters. The behavior of the local population can likewise significantly affect the predictions. Thus, it is not always simple to transpose a generic model to a specific scenario. In addition, it is visible that international models, however complex they may be, do not always represent the reality of a developing country.

Brazil was one of the countries most affected during the first year of the pandemic, in a pre-vaccine era. As of December 2020, it presented the second-highest absolute number of COVID-19 cases in the world (7,213,155 confirmed cases) and the second-highest absolute number of deaths from the disease (186,356 confirmed deaths) [18]. In a country with an enormous territory with continental dimensions and multiple local disparities, including economic, social, and demographic differences, modeling could play an essential role in decision-making and public policies to mitigate and suppress SARS-CoV-2 dispersion.

Although there are a few reviews about mathematical modeling and COVID-19, there is a lack of reviews on models addressing SARS-CoV-2 pandemic impacts in Brazil. Gnanvi et al. [19] conducted a global systematic review of the literature from January 01, 2020, to November 30, 2020, to summarize trends in the modeling techniques used for COVID-19. However, of all 242 papers reviewed, none focused on Brazil, while 18 included Brazil in a general analysis. Kimani et al. [20] thoroughly review infectious disease modeling for SARS-CoV-2 in Africa, which is very enriching to understand the mathematical model’s role in guiding local policy. There are no reviews on Brazil’s mathematical and statistical model approach guiding the pandemic.

Therefore, we ask: What modeling was harbored for COVID-19 in Brazil? What types of models were developed? Which parameters were used? What assumptions were made? What were the questions these models tried to answer? In this article, we intend to overview all the existing published modeling for COVID-19 in Brazil, focusing on the first 18 months of the pandemic. In a scenario of the absence of vaccines and known treatments and the possibility of virus dissemination, we describe the role of mathematical and statistical modeling in guiding public health authorities in Brazil and to what extent this is reflected in the scientific and gray literature.

## Objective

This study aims to review the mathematical and statistical modeling used to evaluate any aspect of the COVID-19 pandemic in Brazil, with emphasis on the first 18 months (between January 01, 2020, and June 2, 2021) when no effective drugs or vaccination were available.

### Research questions

Research questions were defined to reflect the mapping of modeling works and studies developed in Brazil during the COVID-19 pandemic and were the following:

- Which were the studying groups working on COVID-19 modeling during the pandemic in Brazil?
- What were the models being used, considering their structure, type, main outputs, parameters, and programming methods?
- Which were the data sources used in the modeling process?

## Methods

### Protocol and registration

This scoping review was developed in five stages that consisted of the (i) definition of the research question, (ii) elaboration of search strategies, (iii) assessment of study eligibility, (iv) data extraction, and (v) summary of findings. This methodological framework was proposed by Levac et al. [21] (and the methodology of the Joanna Briggs Institute (JBI) [22]. The study report was structured to adhere to the PRISMA extension for scoping reviews (PRISMA-ScR) [23]. A protocol describing the review methods was developed a priori and made available at Open Science Framework [24].

### Eligibility criteria

To be included in this review, studies needed to present COVID-19 or SARS-CoV-2 modeling in Brazil, including infectious disease modeling methods. Peer-reviewed journals papers, gray literature, or database repositories files were included if they were: published between January 01, 2020, and June 2, 2021, written in any language, involved COVID-19 analyses and modeling methodology (including analytical decision models, cohort and Markov models, compartmental models, and individual agent-based modeling), reporting real-world or scenario-based COVID-19 modeling, for Brazil. Papers were excluded if they did not fit the study’s conceptual framework (modeling of non-COVID outcomes) or were not fully available (works not fully retrieved or poster abstracts). Case reports, case series, guidelines/recommendations, letters/perspective/editorials/comments pieces, reviews, and descriptive epidemiological publications were excluded. Studies presenting only mathematical models or software tools but not modeling outputs, projections, results, or works using projections only as examples of the models’ application were excluded. Studies modeling vaccine outputs were also excluded. S1 Table shows all the inclusion and exclusion criteria.

### Information sources

In order to identify the potentially relevant studies, search strategies were applied in MEDLINE (via PubMed) and adjusted for the other databases (Embase and LILACS). Gray literature (papers published but not peer-reviewed) was searched in ArXiv, Medrxiv, Biorxiv, and Open Gray. Repositories accessed were Figshare, Github, Zenodo, and Dryad. Additionally, reference lists of included studies were hand-searched to identify potentially eligible studies. The searches were carried out from May 31 to June 2, 2021. S2 Table shows all the bases accessed and the search strategies applied.

### Search strategy

The search strategy was developed and refined with the help of epidemiologists with experience in systematic review and modelers working jointly with our research team. The search strategies are presented in S2 Table.

### Selection of sources of evidence

The studies were selected per the methodological guidelines of scoping reviews to meet the study’s aims and objectives. References identified were screened in Rayyan [25]. Titles, abstracts, and data repository descriptions identified were read and selected by four independent reviewers (three epidemiologists and one modeler), in pairs, in parallel, who were not blind to the journal titles or the study authors or institutions. Inconsistencies and disagreements were solved by consensus or in discussion with other reviewers. After this first screening, full-text readings of the published and unpublished documents included in the review were conducted. This process was iterative, and the criteria could be updated throughout the selection.

### Data charting process

Two investigators extracted the data independently—one epidemiologist and one modeler. The two reviewers discussed the results and solved disagreements by consensus. The data charting and discussion of results was an iterative process.

### Data items

The framework for data extraction was a priori defined to reflect the research questions mentioned, comprising the studies’ characterization. We gathered information regarding authors, geographic location modeled, modeling period, institutional affiliation of authors, publication timeline, modeling objectives, modeling methods, and main results. The following modeling objectives were considered: analysis of the epidemiological scenario, short- and long-term projections without interventions, and economic analysis. Regarding modeling methods, we focused on (I) Model type (Compartmental, Statistical/Probabilistic, Machine-Learning, Agent-Based/Individual, Mixed; Deterministic or stochastic; Dynamic or static) and structure (number and class of compartments, if applicable); (II) Main objective (specific interventions, short- and long-term future projections, or economic analysis); (III) Source of data inputs used in the model; (IV) Spatial scale for which the estimations have been done; (V) Time frame and time horizon; (VI) Model parameters and data sources for each parameter; (VII) Model output(s); (XIII) Model fitting and calibration approaches; (IX) Sensitivity analysis; (X) Model code availability.

### Critical appraisal of individual sources of evidence

No quality assessment of the studies was applied.

### Synthesis of the results

The results will be presented systematically. Firstly, we will present the results of the searches, screening, and inclusion of studies. Then, a general descriptive analysis will be conducted on the incorporated studies, including publication year and type, country of origin, and types of models used.

The included studies were also classified according to the modeling objective and will be presented according to their scope: analysis of specific interventions, short- and long-term future projections, and economic analysis. A Venn diagram will be presented as each study may belong to more than one category.

Next, a more detailed descriptive analysis will be performed regarding the types of studies and models found. For didactic purposes, we separated compartmental models from all other types of models, and two tables were generated to organize the information.

We will also focus on the parameters found in the articles included in this review and used to run the evaluated models, a descriptive analysis of the presence or absence of fitting and calibration procedures of the models, and the public availability of the used codes.

## Results

**Fig 1:**
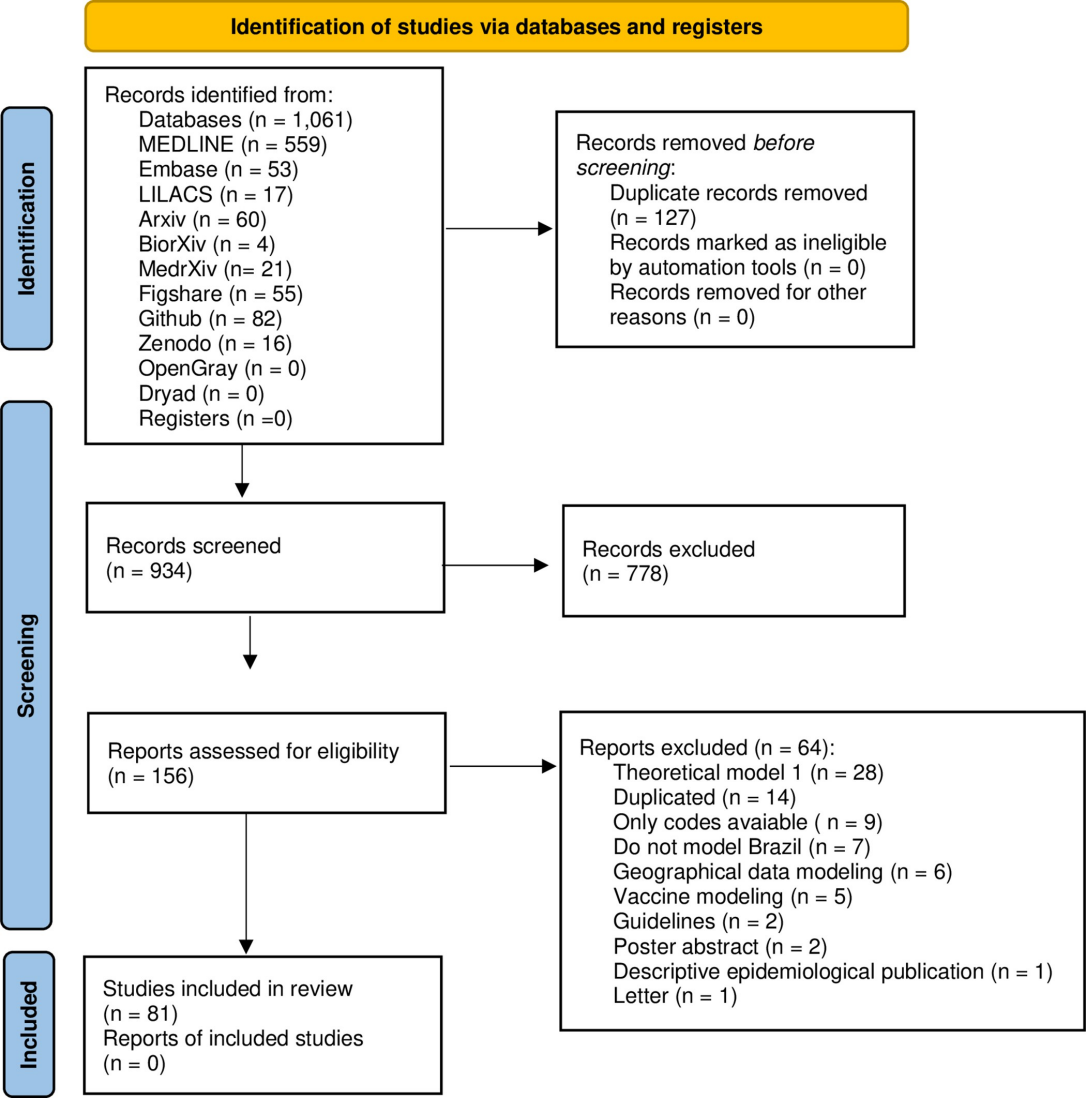
Prisma flow-chart for studies identification, screening, and inclusion.

Electronic and additional searches retrieved 1061 references. After removing duplicates (127), titles and abstracts of 934 references were screened, leading to a selection of 156 full texts. Of those (156) studies that were fully assessed for eligibility, seventy-five (75) were excluded for various reasons, comprising finally 81 studies included in this review (Fig 1). The list of excluded studies at the full-text reading stage and the reasons for exclusion are presented in S3 Table.

Of all the references evaluated, 79.3% (65) correspond to published articles, and 20.7% (17) were publications without a peer-review process (gray literature). For the published articles, the average time between submission and publication was approximately 114 days, with a median of 98 days. The minimum time between submission and publication was seven days, and the maximum time found was 395 days.

Table 1 shows the summary of findings with the date of publication, the origin of the studies, and the types of models used. As for the main institutional affiliation of investigators of the studies, most are Brazilian. The authors of 45 (54.9%) studies were affiliated with Brazilian institutions. Authors from foreign institutions produced sixteen (19.5%) studies without the participation of Brazilian institutions. Twenty-one (25.6%) studies resulted from a collaboration between Brazilian and international institutions. Regarding modeling approach and model type, most were classified as dynamic (71; 87.6%) and deterministic (57; 70.3%). The predominant type of model was compartmental (51; 62.9%). Other types of models found were statistical/probabilistic (18; 22.2%), mixed (8; 9.9%), machine learning (3; 3.7%), and agent-based/individual (1; 1.2%). Mixed models refer to a mixture of two different models combined.

**Table 1:** Summary of findings: Date of publication, the origin of the studies, and types of models.

Works included		81 (100%)
Year of publication		
	2020	59 (72.8%)
	2021	22 (27.2%)
Location of institutions to which authors were affiliated		
	Brazil	45 (55.5%)
	Foreign	15 (18.5%)
	Collaborations	21 (26%)
Type of model		
	Compartmental	51 (62.9%)
	Statistical/Probabilistic	18 (22.2%)
	Machine-Learning	3 (3.7%)
	Agent-Based/Individual	1 (1.2%)
	Mixed	8 (9.9%)

**Fig 2:**
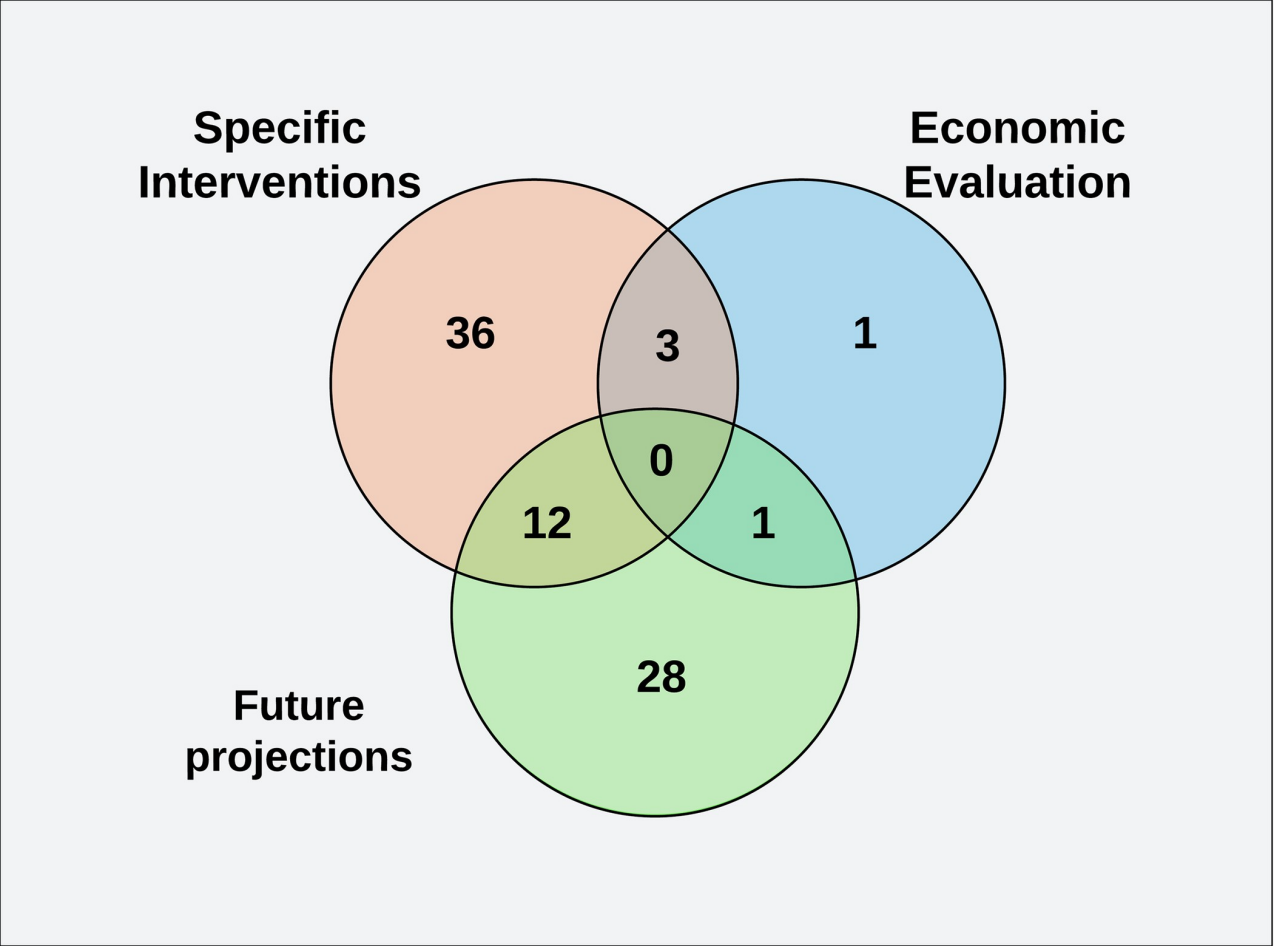
Venn diagram (modeling objectives). The works were classified according to the main objective of the modeling: analysis of specific interventions (with the evaluation of possible intervention measures), short- and long-term predictions (future projections), and economic analyses.

When studies were classified according to the main modeling objectives, most of them analyzed specific interventions (mainly testing interventions such as lockdown, vertical isolation, and closure of commerce and schools), projected short- and long-term epidemiological scenarios with real data, but not testing interventions (future projections), or both. Only a few articles performed economic impact analysis (Fig 2).

When analyzing the reviewed models, as depicted in Table 2, we have identified three compartmentalization patterns based on the disease’s natural history. The majority of the articles differ in whether or not they include a compartment referring to the population exposed to the virus, considered infected but not infectious. Therefore, we have classified compartmental models into two categories: SIR–Susceptible-Infectious-Recovered (15, 29.5%) and SEIR–Susceptible-Exposed-Infectious-Recovered (34, 66.7%). Only two articles (2, 3.8%) also considered the possibility of reinfection, introducing a new compartment for new susceptibles, and were categorized as SEIRS (Susceptible-Exposed-Infectious-Recovered-Susceptible).

**Table 2:** Studies on compartmental modeling, stratified by the natural history of the disease and type of modeling, with details referring to the assumptions, stratifications and subpopulations modeled, geographic area studied, time horizon and the main outputs investigated.

Model	Natural History	Model type	Assumptions	Stratification/ subpopulations	Spatial scale	Time Horizon	Purpose	Main outputs
**SIR**	Do not include exposed/latent compartment; Do not consider reinfections;	Deterministic	**Asymptomatic, mild, moderate and severe disease** [26]; **Asymptomatic and symptomatic** [27–31]; **Government interventions** [30, 32]; **Mobility** [33]; **Healthcare capacity** [26, 29, 33]; **Natural birth and death rates** [34]; **Delays in state and control variables** [28]; **Considered a population response parameter** [29, 31]; **Underreporting** [31];	**Age structured** [33]; **Favelas’ population** [26]; **School and university population** [33];	**Country** [28–36]; **City** [37, 38]; **State** [27, 39]; **Assumed** [26];	**Various** [35]; **200 days** [36]; **80 days** [34, 37] **2 years** [26]; **50 days** [32] **100 days** [28, 33]; **150 days** [27]; **10 days** [38]; **2000 days** [39]; **400 days** [29]; **3 months** [30]; **360 days** [31];	**Future projections** [30, 35–38] **Specific interventions** [26–34, 39] **Economic analyses** [28];	**R0** [33, 35, 37, 39]; **Prediction of the epidemic** [27–38]; **Transmission rate** [32, 37]; **Peak of the epidemics** [36]; **ICU availability** [26, 33] **Symptomatic and asymptomatic** [27, 28, 30, 31] **Isolation effect** [29, 39]; **Hospitalizations** [28]; **Economic evaluation** [28];
		Stochastic	**Government interventions** [40]; **Mobility** [40]; **Healthcare capacity** [40];	**School population** [40];	**City** [40];	**720 days** [40];	**Specific interventions** [40];	**Prediction of the epidemic** [40];
**SEIR**	Exposed compartment included; Do not consider reinfections;	Deterministic	**Asymptomatic and symptomatic** [41–50] **Mild, moderate and critical disease** [46–52] **Healthcare capacity** [45, 48, 51–57]; **Different case-fatality rates** [51, 53] **Vertical social distance policies** [46, 55, 58]; **Underreporting** [42, 56]; **Environmental resevoir** [59]; **Natural birth and death rates** [46, 47, 49, 50, 59, 60]; **Mobility** [44, 51, 52, 59, 61]; **Government interventions** [43, 44, 61, 62]; **Distinct incubation rates for variants** [63]; **Distinct asymptomatic/symptomatic rates for variants** [47]; **Considered limited resources** [45]; **Considered tested and untested population** [50, 64];	**Age structured** [46, 47, 49, 51, 55, 56, 58, 60, 65–67]; **School population** [41];	**Country** [42, 45, 51, 53, 54, 56, 57, 63, 65–69]; **State** [46–50, 52, 58, 59, 61, 64, 70–72]; **City** [41, 43, 55, 60]; **Regional** [44];	**120 days** [65, 69]; **480 days** [71]; **365 days** [54] [47]**; 150 days** [58] [42]; **250 days** [55] [45]; **300 days** [56] [59]; **500 days** [51, 64, 67]**; 30 days** [41]; **180 days** [43, 46]; **60 days** [60, 66]; **3 weeks** [63]; **5 months** [50]; **6 months** [48, 49, 72]; **7 months** [56, 72]; **2 years** [52, 53];	**Future projections** [42, 43, 46, 51, 54, 56, 60, 62, 65, 71, 72] **Specific interventions** [41, 42, 44–50, 52–59, 61, 63, 64, 65–67, 69]; **Economic analyses** [50, 66];	**R0** [41–45, 48–52, 59, 62, 65, 71]; **Prediction of the epidemic** [41–43, 46–55, 57–66, 69, 71, 72]; **Hospitalizations** [48, 51, 55, 60, 66, 67]; **Peak of epidemics** [52, 56, 65]; **Isolation effect** [45, 49, 65, 66]; **Symptomatic and asymptomatic** [42, 43, 45, 50, 62, 67]; **Secondary cases caused by infected children** [41]; **Transmission rate** [44, 45]; **Economic evaluation** [50, 66]; **ICU availability** [48, 56, 57]
		Stochastic	**Natural birth and death rates** [63]; **Mild, severe and critical disease** [73]; **Asymptomatic and symptomatic** [74]; **Considered a population response parameter** [73]; **Mobility** [73]; **Healthcare capacity** [73]; **Underreporting** [74];	**Age structured** [75];	**City** [75]; **Regional** [73]; **Assumed** [74];	**60 days** [75]; **365 days** [73]; **120 days** [74];	**Future projections** [74]; **Specific interventions** [73–75];	**Prediction of the epidemic** [73–75]; **Isolation effect** [73, 75]; **R0** [73] [74]; **Peak of epidemics** [70]; **Hospitalizations** [73]; **ICU availability** [73];
**SEIRS**	Exposed compartment included; Considers reinfections;	Deterministic	**Healthcare capacity** [76]; **Mobility** [76]; **Asymptomatic, symptomatic and hospitalized** [75]; **Considered reinfection by gamma (P1) variant** [77]; **Distinct transmissibility rates for variants** [77];	**Age structured** [77];	**City** [77]; **State** [76];	**365 days** [76]; **3 months** [77];	**Future projections** [77]; **Economic analyses** [76];	**Prediction of the epidemic** [76]; **Hospitalizations** [76, 77]; **ICU availability** [76]; **Transmission rate** [77]; **Economic evaluation** [76];

While many articles vary in the number of compartments presented, ranging from 3 to 9 compartments, we have chosen not to consider the original compartmentalization of each article. This decision is based on the recognition that many of the compartments presented do not represent a differentiation in the dynamics of the disease’s natural history but are compartments created to facilitate the modeling process and the presentation of results. Consequently, works incorporating additional compartments, such as symptomatic, asymptomatic, quarantined, hospitalized, or deceased individuals, have had their specific characteristics addressed in the "assumptions" column.

The studied models significantly differed in the considered assumptions, highlighting their substantial diversity. Many of these models (21, 41.2%) accounted for the possibilities of varying degrees of disease with different specific transmissibilities and mortalities (asymptomatic, symptomatic, mild, moderate, or severe disease). Furthermore, many considered governmental non-pharmacological interventions (7, 13.7%) and healthcare service capacity (15, 29.5%). In contrast, only a few models took into account underreporting (4, 7.8%) or testing capacity (3, 5.9%) during the modeling process.

Regarding the stratifications or modeled subpopulations, we identified only three approaches adopted by the studies. Among those utilizing this methodology, age-structured models were the most commonly employed (29.5%). Additionally, we found a few studies that modeled specific populations, such as schools and universities (5.9%), along with a solitary study that focused its modeling on the populations residing in favelas (1.9%).

The compartmental models were mainly used to predict the epidemic behavior for larger spatial scales, such as countries (22, 43.1%) and states (16, 31.4%), albeit a few articles also made predictions for more minor spatial scales, such as cities (10, 19.6%) and regions (3, 5.9%).

Among the outputs, estimations of the R0 (21, 41.2%) and transmission rates (5, 9.8%) or projections regarding the course of the epidemic (43, 84.3%) and anticipation of the transmission peak (5, 9.8%) figured as major, especially at the beginning of the crisis. However, considering that many articles made more than a unique prediction, several other outputs were forecasted through the period, such as the isolation/quarantine effect on epidemic transmission (8, 15.7%), symptomatic and asymptomatic persons (10, 19.6%), ICU hospital facilities required (7, 13.7%) and hospitalizations (9, 17.6%), secondary cases caused by infected children (1, 1,9%), and the economic effects of the pandemic (4, 7.8%).

The other models identified were agent-based/individual (1, 3.3%), machine learning (3, 10%), statistical/probabilistic (18, 60%), and mixed (8, 26.7%), and they are listed in Table 3, also stratified by type of modeling, spatial scale, time horizon, and main outputs. Here, one can see the various models applied to different modeling objectives and the investigation of desired outputs. Most studies used stochastic models to predict epidemics on a country scale, although some works also modeled states and cities. A range of statistical and probabilistic models was utilized (Bayesian, ARIMA, and many others), but agent-based/individual and machine-learning models were restricted to a few. Concerning mixed models, they all represented a mixture of a compartmental and a different type of model (Bayesian, Markov chain, Agent-based/individual, e.g.).

**Table 3:** Studies on COVID-19 models other than compartmental models, stratified by the type of modeling, spatial scale, time horizon, and main outputs.

Models	Model type	Spatial Scale	Time Horizon	Main outputs	References
**Agent-based/Individual**	Stochastic	**Assumed**	**365 days**	**R0; Prediction of epidemic; Symptomatic and asymptomatic; Hospitalizations; Isolations effects; Herd immunity; Case-fatality rate; ICU availability; Economic evaluation**	[78]
**Machine Learning**					
Long Short-Term Memory for Data Training (LSTM) with Deep Learning	Stochastic	**Country**	**5 months** [88]; **8 months** [89];	**Prediction of epidemic**	[79, 80]
Long Short-Term Memory (LSTM), Gated Recurrent Unit (GRU), Convolutional Neural Network (CNN), Multivariate Convolutional Neural Network (MCNN);	Stochastic	**Country**	**40 days**	**Prediction of epidemic**	[81]
**Statistical/Probabilistic**					
Bayesian Model	Stochastic	**Country**	**4 months** [91]**; NA** [92];	**Prediction of epidemics, Peak of epidemics, ICU availability** [91]; **Mortality risks and infection risk** [92];	[82, 83]
Boltzmann Function Regression Analysis	Stochastic	**State**	**3 months** [93]**; 150 days** [94];	**Prediction of epidemic**	[84, 85]
Generalized Linear Model (GLM) Prospective Space–time Scan Statistic	Stochastic	**City** [95]; **State** [96];	**6 months** [95]; **NA** [96];	**Geographical analyses and relative risks for COVID-19**	[86, 87]
Autoregressive Integrated Moving Average (ARIMA)	Stochastic	**State**	**6 days**	**Prediction of the epidemic**	[88]
Weibull Distribution	Stochastic	**Country**	**200 days**	**Prediction of the epidemic**	[89]
Skew-normal Distributions	Stochastic	**Country**	**50 days**	**Transmission rate; Prediction of the epidemic; Peak of the epidemic; Mortality rate.**	[90]
Logistic Growth Model	Stochastic	**State**	**250 days**	**Prediction of the epidemic**	[91]
Discrete-time Model	Stochastic	**Country**	**14 days**	**Prediction of the epidemic**	[92]
Richards Growth Model (RGM)	Stochastic	**Country**	**100 days**	**Prediction of the epidemic**	[93]
Holt-Winters Exponential Model	Stochastic	**Country**	**7 days**	**Prediction of the epidemic**	[94]
Gompertz Exponential Model	Stochastic	**Country**	**7 days**	**Prediction of the epidemic; R0.**	[95]
Lifshitz Scaling Diffusion Equation	Stochastic	**Country**	**200 days**	**Prediction of the epidemic**	[96]
Finite Chains of Recurrent Sequences	Stochastic	**Country**	**365 days**	**Prediction of the epidemic**	[97]
Probabilistic	Stochastic	**Country**	**NA**	**Isolation effects; Probability of outbreak; Social vulnerability.**	[98]
Various	Stochastic	**Country**	**30 days**	**Prediction of the epidemic; R0.**	[99]
**Mixed**					
Compartmental and Agent-Based/Individual	Stochastic	**Assumed** [39]; **Country** [76, 78];	**60 days** [39]; **300 days** [76]; **3000 days** [78];	**Prediction of the epidemic** [39, 76, 78]**; Hospitalizations** [39, 76]**; Economic evaluation** [39]**; ICU beds availability** [76, 78]**; R0** [78];	[70, 100, 101]
Compartmental and Non-Linear Model Predictive Control (NMPC)	Stochastic	**Country**	**Various**	**Prediction of epidemic, symptomatic and asymptomatic; R0.**	[102]
Compartmental and Markov chain	Stochastic	**Country**	**4 months**	**Prediction of the epidemic; Symptomatic and asymptomatic; Hospitalizations; ICU bed availability.**	[103]
Compartmental and Bayesian model	Stochastic	**Country**	**10 months**	**Prediction of the epidemic; R0.**	[104]
Compartmental and Neural Network Module (Machine Learning)	Stochastic	**Country**	**60 days**	**Prediction of the epidemic; Isolation effects.**	[105]
Compartmental and Metapopulation models	Stochastic	**State**	**30 days**	**Prediction of epidemic**	[106]

Considering that parameters directly influence the performance of the models and the obtained outputs, an assessment of the parameter sources was conducted. Parameters were categorized as follows: extracted from literary sources, obtained from government and non-governmental data sources, and directly used in the model without any new adjustments (assumed); calculated and adjusted from secondary data sources (estimated from secondary data); or estimated from the evaluated model itself (modeled). The main parameters evaluated, their sources, and the references used are listed in Table 4. All data sources used by the evaluated articles were assessed and are listed in S4 Table.

**Table 4:** Models’ parameters, their primary sources, and references.

Parameters	Definition	Number of studies utilizing the parameter in the model	Source		
			Estimated from secondary data	Modeled	Assumed
**R0**	The average number of secondarily infected persons infected by one primary infected patient during the infectious period.	48	7 (15%)	30 (62%)	11 (23%)
			[30, 46, 47, 49, 51, 62, 75]	[27, 28, 33, 35–39, 41–45, 48, 51, 54, 59, 61, 65, 67, 74, 78, 95, 99, 101, 102, 104, 105, 107–109]	[55, 59, 60, 64, 69, 71, 76, 82, 96, 106]
**Re**	The effective reproduction number	29	3 (10%)	25 (86%)	1 (4%)
			[49, 63, 75]	[36, 38, 41, 43, 44, 46–48, 51, 52, 59, 61–64, 71, 73, 78, 99, 101–103, 107–109]	[76]
**Serial Interval**	The time from the onset of symptoms in the primary case to the onset of symptoms in the secondary case	3	1 (50%)		1 (50%)
			[63]		[82]
**Incubation Period**	The time between infection and onset of symptoms	43	4 (9%)	2 (5%)	37 (86%)
			[42, 54, 56, 73]	[72, 108]	[26, 28, 40, 43, 44–49, 51–53, 55, 58–63, 64–66, 70, 74, 76–78, 82, 92, 100–103, 106, 107, 109]
**Infectious Period**	The time interval during which the infected individuals could transmit the disease to any susceptible contacts	41	5 (12%)	5 (12%)	31 (76%)
			[28, 29, 42, 56, 73]	[31, 36, 48, 104, 108]	[26, 27, 30, 33, 41, 43, 46, 47, 49, 51, 52, 55, 58–61, 64, 65, 69, 70, 72, 74, 75, 77, 82, 92, 98, 100, 101, 103, 109]
**Case fatality rate**	The proportion of people who die among all individuals who have been diagnosed.	54	15 (28%)	26 (48%)	13 (24%)
			[28, 30, 32–34, 45, 46, 56, 57, 59, 64, 66, 73, 75, 107]	[27, 29, 31, 37, 38, 44, 47, 49, 51–54, 61–63, 71, 72, 77, 83, 89, 90, 93, 99, 102, 104, 109]	[26, 41, 48, 55, 60, 67, 71, 76, 78, 82, 92, 100, 103]
**Proportion of suceptible**	The proportion of a population that is susceptible to COVID	72	53 (74%)	9 (12%)	10 (14%)
			[27–34, 36, 37, 39, 40, 43–49, 51, 52, 54–58, 60–63, 64–67, 69, 71, 73, 75–77, 82, 83, 85, 86, 90, 91, 93, 99, 101, 104, 105–107]	[35, 38, 41, 51, 53, 72, 74, 107, 108]	[26, 42, 59, 70, 78, 89, 92, 98, 102, 103]
**Mobility**	The movement of people within a population.	14	5 (36%)		9 (64%)
			[43, 59, 61, 73, 106]		[33, 40, 42, 52, 76, 78, 98, 100, 102]
**Contact rate**	The average number of appropriate contacts through which disease transmission occurs per individual per unit of time	37	6 (16%)	17 (46%)	14 (38%)
			[27, 29–31, 41, 54]	[33, 38, 47, 51, 57, 63, 69–71, 74, 100, 102, 103, 105, 107–109]	[26, 36, 40, 48, 49, 55, 58–60, 66, 75–77, 107]
**Birth/death rate**	Birth and natural death	10	1 (10%)		9 (90%)
			[59]		[28, 34, 41, 46, 47, 60, 75, 101, 107]
**Healthcare capacity**	Amount of healthcare facilities available at the time of modeling	24	4 (17%)	1 (4%)	19 (79%)
			[26, 52, 55, 73]	[45]	[29, 33, 40, 48, 51, 53, 54, 56, 57, 70, 76, 78, 82, 83, 90, 98, 101–103]

Regarding the model fitting approach, sixty-one (61; 74,4%) studies reported having some form of calibration or adjustment using real data, while only 10 (12,1%) explicitly stated not using any specific method for this purpose, and in 11 (13,5%) cases, fitting methods were not possible or not reported. As calibration plays a pivotal role in the modeling process, particularly with the level of reliability required for COVID-19 decision-making, approximately 70% of the articles provided details of their calibration parameters and the data employed in the process. Notably, about 30% of the reviewed articles omitted any reference to their fitting step. Among the articles that did discuss model calibration, we compiled in S5 Table the key parameters that were adjusted, as well as a comprehensive list of the primary data sources used for calibration.

Sensitivity analyses were conducted in only 16 (19.7%) studies; in 66 (81.5%) studies, they were either not performed or not reported. Model codes were publicly available in only 30 (37%) of the evaluated studies, whereas in 52 (64.1%) studies, it was impossible to access the codes.

## Discussion

The substantial number of mathematical and statistical modeling studies in this review may suggest models played an important role in supporting decision-making in Brazil’s first year of the COVID-19 pandemic. Like in other countries, research groups in statistics and predictive modeling directed their efforts toward studying SARS-CoV-2. Additionally, new groups were formed, bringing together modelers, epidemiologists, and infectious disease experts to work collaboratively [110–112].

Our results are a reflection of this scenario. In a search limited to the first 18 months of the pandemic, we included 81 articles (published in scientific journals or available in non-peer-reviewed databases) that analyze any aspect of COVID-19 using a modeling approach. The studies were predominantly Brazilian or conducted in collaboration between Brazilian and foreign entities. This fact demonstrates a clear interest among Brazilian researchers in generating scientific evidence to strengthen the fight against the epidemic within the country. Also, Brazil was a considered topic for international institutions without the involvement of Brazilian universities or researchers, reflecting an interest in Brazil as a subject of study: an enormous country with a continental dimension, local disparities, social inequities, heterogeneous population, and diverse public health management regarding the pandemic.

An important aspect to highlight is the elapsed time between article submission and its publication, with an average of 114 days and a median of 98 days, but with articles taking up to a year to be published. All the difficulties encountered in reviewing an original manuscript are justifiable, and the elapsed time is understandable; however, this does not apply to a situation like a pandemic. Thus, several studies were published in gray literature without peer review in this emergency to make the results publicly available and provide a basis for public health decision-making. It should be noted that several articles were excluded from the final selection due to duplicates, meaning that they were published as preprints and were also already published in scientific journals with appropriate peer review at the time of this review.

Our results demonstrate that the majority of models utilized were dynamic, deterministic, and compartmental. These models are the most commonly employed in infectious diseases and enable the simulation of transmission dynamics in a fully susceptible population, as was the case with the introduction of SARS-CoV-2 in Brazil [113]. These models were so crucial worldwide that early in the pandemic, several authors and institutions published guidelines on how to structure a model, providing instructions on basic frameworks, differences between models, and characteristics that can be implemented to enhance their performance. For instance, Lu Tang et al. [114] published a review in 2020 that specifically focused on guiding SARS-CoV-2 modeling, highlighting these types of models, and providing guidance on the utilization of key parameters, along with supplying code for implementation in software packages.

Other types of models are equally important and were also found. These included statistical, probabilistic, machine learning, and agent-based/individual models. Generally, these types of models often require more robust databases to yield better results. However, this may not be feasible at the beginning of a pandemic due to the inherent nature of the natural history of infection spread and the laborious process of collecting, making publicly available, and ensuring the robustness and accessibility of the databases. Another significant point is that these models rely on historical data series, generating outputs heavily based on past events. As a result, they may lose sensitivity to population behavior changes, the implementation of non-pharmaceutical interventions over time, or the occurrence of new waves that could not be detected prior to an initial decline.

This was the case in the only study that employed a purely agent-based/individual-based approach. Staffini et al. [78] developed a model to assess different political approaches for containing COVID-19 in four selected countries (Brazil, Sweden, Italy, and Germany). Brazil was chosen precisely because it did not adopt nationwide control measures, despite having one of the highest death tolls in the world. The model examined virus spread through the interaction between healthy and infected agents over time, considering the government actions documented in each country’s official documents. However, the simulations were conducted with a fixed population of 1000 agents and a time span of 365 days, indicating that this type of study serves primarily for a theoretical evaluation of epidemic dynamics and is less suitable for guiding short- and medium-term public policies. Furthermore, this type of model was only capable of predicting a single curve, with clear ascending and descending periods, without forecasting waves of contagion and transmission.

These findings are directly related to the ultimate objective of each study and modeling effort. Parametrized models enable us to test various situations and envision scenarios crucial for informing public health policies. Therefore, it is unsurprising that most examined articles were classified as *specific interventions*. However, dynamic and compartmental models were also utilized to project an epidemiological scenario without interventions (*future projections*), with parameters based on the epidemiological data available during model execution.

Notably, a limited number of studies are dedicated to conducting an economic analysis of the COVID-19 situation in Brazil. The fact that Brazil possesses a comprehensive, universal, and free public healthcare system may have discouraged research in the field of economic analyses of the pandemic. However, it is also worth considering the complexity of the parameters to be used in the economic analysis of the studied models, a condition that may have been considered in a context requiring rapid production of information for decision support.

We can observe a significant variability in the complexity of the implemented models. If we look at compartmental models, we can see that they can exhibit more or less complex structures, not only reflected by the number of compartments ranging from 3 to 9, but primarily by the natural history of the disease, assuming or not an exposed compartment, or even considering the existence of new susceptibles to reinfections. However, other characteristics can increase the model’s complexity, aiming to bring it closer to reality. These include population stratification by age, parameter variation using the gamma and log-normal distributions, or incorporating social, demographic, and geographical data, translating real-world aspects into a more sensitive parameterization with bolder assumptions. We can observe this in several studies. For example, Almeida et al. [75], despite using only five compartments, and considering an exposed compartment, managed to bring shades of reality to the model by calculating the R_0_ for each modeled City based on real data and conducting a cluster analysis of cities using demographic, social, and climatic characteristics of each studied unit. On the other hand, in addition to employing a more complex model with nine compartments, Amaku et al. [50] dissected numerous parameters, assumed or fitted, and performed an additional fitting procedure for the model’s outputs.

Furthermore, the other non-compartmental models studied are naturally more complex. As mentioned, they rely on a robust database and generally require more advanced technological tools for implementation. We found only three studies that exclusively used machine learning techniques. Yogesh Gautam et al. [79] employed transfer learning for Long Short-Term Memory for Data Training (LSTM) networks but had to utilize Italian and American data to model other countries such as Brazil, India, and Nepal. Igor Gadelha Pereira et al. [80] also utilized LSTM, but with Deep Learning, and initially obtained disappointing results but concluded that it is a path for future studies. On the other hand, Khondoker Nazmoon Nabi et al. [115] employed a series of automated methodologies, including LSTM, Gated Recurrent Unit (GRU), Convolutional Neural Network (CNN), and Multivariate Convolutional Neural Network (MCNN). However, they obtained unreliable results, as seasonal patterns and periodic intervals could not be captured.

An understudied aspect that has been determinant in the evolution and spread dynamics of COVID-19 in Brazil is the profound social, economic, and territorial inequalities existing in the country. Translating the social determinants of the health-disease process into mathematical models is not easy; besides models often neglecting this bias, this fact is still poorly discussed, and necessary caveats are not frequently addressed.

An interesting study by Flávio C. Coelho et al. [98] employed a probabilistic model to identify areas in Brazil with higher vulnerability to COVID-19 regarding the risk of new case introduction, sustained transmission, and social vulnerability. It is noteworthy that this study provided one of the most comprehensive characterizations of social vulnerability, including measures such as infant mortality, life expectancy, GINI index, the proportion of the population below the poverty line, proportion of urban and rural populations, access to safe water, sewage system, and electricity. The findings were consistent with what has been observed throughout the pandemic regarding morbidity and mortality in vulnerable areas and deserve recognition, as in Brazil, it is impossible to discuss infectious diseases without considering social differences and individual and collective vulnerabilities.

Another study meritorious of attention is the one conducted by VP Rodrigues and colleagues [26], which provides a comprehensive analysis of the pandemic, focusing on the population living in Brazilian favelas. The article proposes using system dynamics to assess the effects of virus spread in the population and to test bold interventions, such as the temporary relocation of the residents, the provision of hygiene supplies, emergency sanitation structures, and the expansion of intensive care units. This was the only article that considered modeling this specific type of situation. Despite the difficulty in parameterization and dependence on assumptions, it is demonstrated that having this perspective is essential and feasible. Zelner et al. [116] extensively review the gaps in accounting social and structural factors to infection models and suggest various ways to include social inequities in modeling.

An important finding worth highlighting, though somewhat expected, is the scarcity of studies considering the presence of variants of concern or interest in the proposed models. Since these studies were conducted early in the pandemic, the presence of variants was not well-known for a considerable period. When their presence was detected, estimating the parameters and comprehending the reinfection process remained challenging.

Celaschi et al. [63] evaluated the impacts of the circulation of lineage B of the original virus in 2020 without assuming reinfections but by proposing different incubation rates, fractions of symptomatic infected individuals, and a time-varying reproduction number. In 2021, Yang et al. [47] and Coutinho et al. [77] modeled the impacts of the gamma variant on the population. Yang. [47] assumed that transmissibility remained unchanged, but his model considered that more virulent variants increase the likelihood of symptomatic illness, thus manifesting the presence of the variant through the asymptomatic/symptomatic ratio. Similar to Celaschi [63], he also did not allow for the possibility of reinfection. Meanwhile, Coutinho et al. [77] conducted a study using actual Brazilian epidemiological surveillance data and, employing a model that accommodates reinfections, estimated both transmissibility and reinfection rate by Brazil’s gamma variant (P1).

When analyzing the studies in general, it can be observed that many groups worked with similar objectives and methods, using common data sources and generating outputs that could have been analyzed together better to understand transmission scenarios and adjustment of predictive models. At times, complementary studies are not discussed, even though they could complement each other. The lack of a national modeling network and a forum for modeling groups may explain the redundant works and the low intersection of complementary studies. The absence of a common thread in this scientific aspect is not surprising, as it is also influenced by the Federal Government’s overall decentralization and lack of planning concerning all other fronts of pandemic control. The decision-making role of the 26 Federative States and the Federal District of Brasilia and the decentralization of local decisions at the municipal level also reflects in the generation of scientific evidence by local technical groups, universities, and independent researchers.

Our review stands out for conducting an extensive and detailed search of the mathematical models used in Brazil’s global emergency caused by COVID-19. Among its strengths, we emphasize the description of the models, their assumptions, the parameters used, their sources, and the calibration and fitting processes carried out during the pandemic. This review looks to the past in order to shape the future. It revisits the modeling process from the perspective of identifying gaps and facilitating work and communication in a potential future pandemic. However, this review has limitations, such as the difficulty in comparing the models, the absence of a quality analysis of the evaluated works, and the inability to assess and compare modeling results. Furthermore, there are no other reviews of mathematical models for COVID-19 in Brazil, which makes it impossible to compare our results with different working groups or methodologies employed.

This review demonstrates that mathematical modeling has been a valuable tool for combining efforts to combat the COVID-19 pandemic and can be a great ally in addressing future local or global epidemics. To achieve this, it is crucial to emphasize the need for transparency in model development, robustness in the data sources used to parameterize compartmental models and to estimate probabilistic and automated models. Additionally, guidance should be provided for communicating results and fostering sharing among modeling groups, society, and public health policymakers.

## Supporting information

S1 Table Inclusion and exclusion criteria. To be included in this review, studies needed to present COVID-19 or SARS-CoV-2 modeling in Brazil, including infectious disease modeling methods. Peer-reviewed journals papers, gray literature, or database repositories files were included if they were: published between January 01, 2020, and June 2, 2021, written in any language, involved COVID-19 analyses and modeling methodology (including analytical decision models, cohort and Markov models, compartmental models, and individual agent-based modeling), reporting real-world or scenario-based COVID-19 modeling, for Brazil. Papers were excluded if they did not fit the study’s conceptual framework (modeling of non-COVID outcomes) or were not fully available (works not fully retrieved or poster abstracts). Case reports, case series, guidelines/recommendations, letters/perspective/editorials/comments pieces, reviews, and descriptive epidemiological publications were excluded. Studies presenting only mathematical models or software tools but not modeling outputs, projections, results, or works using projections only as examples of the models’ application were excluded. Studies modeling vaccine outputs were also excluded.
(DOCX)

S2 Table Search strategy for COVID-19 models in Brazil, in a pre-vaccine era. Search performed from May 31 to June 2, 2021. In order to identify the potentially relevant studies, search strategies were applied in MEDLINE (via PubMed) and adjusted for the other databases (Embase and LILACS). Gray literature (papers published but not peer-reviewed) was searched in ArXiv, Medrxiv, Biorxiv, and Open Gray. Repositories accessed were Figshare, Github, Zenodo, and Dryad. Additionally, reference lists of included studies were hand-searched to identify potentially eligible studies. The searches were carried out from May 31 to June 2, 2021.
(DOCX)

S3 Table Excluded studies and reasons for exclusion after full-text reading. Electronic and additional searches retrieved 1061 references. After removing duplicates (127), titles and abstracts of 934 references were screened, leading to a selection of (156) full texts. Of those (156) studies that were assessed for eligibility, seventy-five (75) were excluded for various reasons, comprising finally 81 studies included in this review. The list of excluded studies at the full-text reading stage and the reasons for exclusion are presented in S3 Table.
(DOCX)

S4 Table Data sources assessed for the models’ parameters. Considering that parameters directly influence the performance of the models and the obtained outputs, an assessment of the parameter sources was conducted. Parameters were categorized as follows: extracted from literary sources, obtained from government and non-governmental data sources, and directly used in the model without any new adjustments (assumed); calculated and adjusted from secondary data sources (estimated from secondary data); or estimated from the evaluated model itself (modeled). The main parameters evaluated, their sources, and the references used are listed in Table 4 (main text). All data sources used by the evaluated articles were assessed and are listed in S4 Table.
(DOCX)

S5 Table Reporting of calibration parameters and primary data sources. As calibration plays a pivotal role in the modeling process, particularly with the level of reliability required for COVID-19 decision-making, approximately 70% of the articles provided details of their calibration parameters and the data employed in the process. Notably, about 30% of the reviewed articles omitted any reference to their fitting step. Among the articles that did discuss model calibration, we compiled in S5 Table the key parameters that were adjusted, as well as a comprehensive list of the primary data sources used for calibration.
(DOCX)

S1 File (XLSX)
