# Supplementary material for: Dynamic transmission modeling of COVID-19 to support decision-making in Brazil: A scoping review in the pre-vaccine era
Source: PLOS Glob Public Health. 2023 Dec 13;3(12):e0002679. doi: 10.1371/journal.pgph.0002679 (PMC10718415; doi:10.1371/journal.pgph.0002679)
Supplement: S3 Table — Electronic and additional searches retrieved 1061 references. After removing duplicates (127), titles and abstracts of 934 references were screened, leading to a selection of (156) full texts. Of those (156) studies that were assessed for eligibility, seventy-five (75) were excluded for various reasons, comprising finally 81 studies included in this review. The list of excluded studies at the full-text reading stage and the reasons for exclusion are presented in S3 Table. (DOCX) [file pgph.0002679.s003.docx]

**Excluded studies**

**S3 Table: Excluded studies and reasons for exclusion after full-text reading.**

| **ID number** | **Author** | **Year** | **Reason of exclusion** |
| --- | --- | --- | --- |
| 8  9 | M. Amaku[1]  L. L. Lima [2] | 2020  2021 | Duplicated  Do not model Brazil |
| 12 | K.E. ArunKumar[3] | 2021 | Theoretical model |
| 13 | F.T. Fernandes [4] | 2021 | Theoretical model |
| 17 | M. Al‑Raeei [5] | 2020 | Theoretical model |
| 19 | M.A. Amaral [6] | 2020 | Do not model Brazil |
| 20 | C.M.C.B Fortaleza[7] | 2020 | Geographical data modeling |
| 25 | A. M. C. H. Attanayake [8] | 2020 | Theoretical model |
| 26 | P.M.M. Bermudi [9] | 2021 | Geographical data modeling; |
| 27 | Y. Tang [10] | 2020 | Letter |
| 29 | S.L.T. de Souza [11] | 2021 | Do not model Brazil |
| 37 | P.S. Peixoto [12] | 2020 | Geographical data modeling |
| 38 | K.N. Nabi [13] | 2020 | Theoretical model |
| 41 | T. Daghriri[14] | 2021 | Do not model Brazil |
| 43 | B.R.G.M. Couto [15] | 2020 | Not retrieved (Poster abstract) |
| 45 | J.A. Cohen[16] | 2021 | Vaccine modeling |
| 48 | N. Wu [17] | 2020 | Theoretical model |
| 49 | M. Stoddard [18] | 2021 | Vaccine modeling; |
| 53 | M. Amaku [1] | 2020 | Duplicated |
| 54 | O.P. Neto [19] | 2021 | Duplicated |
| 55 | W. Lyra[20] | 2020 | Duplicated |
| 56 | H.M. Yang [21] | 2020 | Theoretical model |
| 58 | B. Nash [22] | 2021 | Theoretical model |
| 60 | J.F. Oliveira[23] | 2020 | Duplicated |
| 62 | M. Amaku[24] | 2021 | Duplicated |
| 63 | G.B. Libotte [25] | 2021 | Theoretical model |
| 64 | F. Pazos[26] | 2020 | Theoretical model |
| 66 | N. Crokidakis[27] | 2020 | Duplicated |
| 70 | P.M.C.L. Pacheco[28] | 2020 | Duplicated |
| 71 | J. Thompson[29] | 2021 | Do not model Brazil |
| 74 | S. Dias[30] | 2021 | Do not model Brazil |
| 75 | A.S. Melo[31] | 2021 | Geographical data modeling |
| 77 | S.M. Moghadas[32] | 2021 | Vaccine modeling |
| 79 | N.R. Faria[33] | 2021 | Descriptive epidemiological publication |
| 81 | M.B. Braga[34] | 2021 | Theoretical model |
| 83 | D.C.S. Gomes[35] | 2021 | Theoretical model |
| 84 | D. Efimov[36] | 2021 | Theoretical model |
| 88 | M.S. Aronna[37] | 2021 | Do not model Brazil |
| 90 | P.H. Borghi[38] | 2021 | Theoretical model |
| 91 | J.A.M. Gondim[39] | 2021 | Theoretical model |
| 93 | S. Mac[40] | 2021 | Guideline |
| 94 | R. Aguas[41] | 2020 | Guideline |
| 95 | K. Friston[42] | 2020 | Theoretical model |
| 96 | L.A. Andrade[43] | 2020 | Geographical data modeling |
| 101 | I.E. Kayral[44] | 2020 | Theoretical model |
| 103 | D.S. Gomes[45] | 2020 | Geographical data modeling; |
| 105 | B.B. Hazarika[46] | 2020 | Theoretical model |
| 108 | A.K. Sahai[47] | 2020 | Theoretical model |
| 109 | H.M. Yang[48] | 2020 | Duplicated |
| 112 | P. Wang[49] | 2020 | Theoretical model |
| 121 | C.E.F. Starling[50] | 2020 | Not retrieved (Poster abstract) |
| 122 | H. Ankaral[51] | 2020 | Theoretical model |
| 124 | D.C.P. Jorge[52] | 2020 | Duplicated |
| 125 | G. Gonzalez-Parra[53] | 2021 | Theoretical model |
| 127 | Pereira [54] | 2021 | Duplicated |
| 129 | M.S. Aronna[37] | 2021 | Duplicated |
| 130 | A.C.S de Oliveira[55] | 2020 | Theoretical model |
| 133 | K.N. Nabi[13] | 2020 | Duplicated |
| 137 | C.M. Batistela[56] | 2021 | Vaccine modeling |
| 138 | A.A. Batista[57] | 2020 | Theoretical model |
| 139 | T. Rodrigues[58] | 2020 | Theoretical model |
| 140 | M.J. Lazo[59] | 2020 | Theoretical model |
| 142 | M.M. Morato[60] | 2020 | Theoretical model |
| 144 | O.P. Neto[19] | 2021 | Duplicated |
| 146 | R.E.R. Gonzales[61] | 2020 | Theoretical model |

**References**

1. Amaku M, Covas DT, Coutinho FAB, Neto RSA, Struchiner C, Wilder-Smith A, et al. Modelling the test, trace and quarantine strategy to control the COVID-19 epidemic in the State of São Paulo, Brazil. MedRxiv 2020. doi:10.1101/2020.12.02.20242743.

2. Lima LL, Atman APF. Impact of mobility restriction in COVID-19 superspreading events using agent-based model. PLoS One 2021;16:1–17. doi:10.1371/journal.pone.0248708.

3. ArunKumar KE, Kalaga D V, Sai Kumar CM, Chilkoor G, Kawaji M, Brenza TM. Forecasting the dynamics of cumulative COVID-19 cases (confirmed, recovered and deaths) for top-16 countries using statistical machine learning models: Auto-Regressive Integrated Moving Average (ARIMA) and Seasonal Auto-Regressive Integrated Moving Avera. Appl Soft Comput 2021;103:107161. doi:10.1016/j.asoc.2021.107161.

4. Fernandes FT, de Oliveira TA, Teixeira CE, Batista AF de M, Dalla Costa G, Chiavegatto Filho ADP. A multipurpose machine learning approach to predict COVID-19 negative prognosis in São Paulo, Brazil. Sci Rep 2021;11:3343. doi:10.1038/s41598-021-82885-y.

5. Al-Raeei M. Numerical simulation of the force of infection and the typical times of SARS-CoV-2 disease for different location countries. Model Earth Syst Environ 2022;8:1443–8. doi:10.1007/s40808-020-01075-3.

6. Amaral MA, Oliveira MM de, Javarone MA. An epidemiological model with voluntary quarantine strategies governed by evolutionary game dynamics. Chaos Solitons Fractals 2021;143:110616. doi:10.1016/j.chaos.2020.110616.

7. Branco Fortaleza CMC, Guimarães RB, de Castro Catão R, Ferreira CP, de Almeida GB, Vilches TN, et al. The use of health geography modeling to understand early dispersion of COVID-19 in São Paulo, Brazil. PLoS One 2021;16:1–14. doi:10.1371/journal.pone.0245051.

8. Attanayake AMCH, Perera SSN. Forecasting COVID-19 Cases Using Alpha-Sutte Indicator: A Comparison with Autoregressive Integrated Moving Average (ARIMA) Method. Biomed Res Int 2020;2020. doi:10.1155/2020/8850199.

9. Bermudi PMM, Bermudi M, Lorenz C, Souza B, Aguiar D, Failla A, et al. Spatiotemporal ecological study of COVID-19 mortality in the city of S˜ao Paulo, Brazil: Shifting of the high mortality risk from areas with the best to those with the worst socio-economic conditions. Travel Med Infect Dis 2021.

10. Tang Y, Serdan TDA, Masi LN, Tang S, Gorjao R, Hirabara SM. Epidemiology of COVID-19 in Brazil: using a mathematical model to estimate the outbreak peak and temporal evolution. Emerg Microbes Infect 2020;9:1453–6. doi:10.1080/22221751.2020.1785337.

11. Souza SLT De, Batista AM, Caldas IL, Iarosz KC. Dynamics of epidemics: Impact of easing restrictions and control of infection spread. 2020.

12. Peixoto PS, Marcondes D, Peixoto C, Oliva SM. Modeling future spread of infections via mobile geolocation data and population dynamics. An application to COVID-19 in Brazil. PLoS One 2020;15:1–23. doi:10.1371/journal.pone.0235732.

13. Nabi KN. Forecasting COVID-19 pandemic: A data-driven analysis. Chaos, Solitons and Fractals 2020.

14. de Melo GC, Duprat IP, de Araújo KCGM, Fischer FM, de Araújo Neto RA. Prediction of cumulative rate of covid-19 deaths in Brazil: A modeling study. Rev Bras Epidemiol 2020;23:1–11. doi:10.1590/1980-549720200081.

15. Couto BRGM, Starling CEF. 433. Mathematical Modeling of COVID-19 Transmission by a k Phases SEIR Model. Open Forum Infect Dis 2020;7:S283-5. doi:10.1093/ofid/ofaa439.627.

16. Cohen JA, Stuart RM, Núñez RC, Wagner B, Chang S, Rosenfeld K, et al. Mechanistic modeling of SARS-CoV-2 immune memory, variants, and vaccines. MedRxiv 2021:2021.05.31.21258018.

17. Wu N, Ben X, Green B, Rough K, Venkatramanan S, Marathe M, et al. Predicting onset of COVID-19 with Mobility-Augmented SEIR Model. MedRxiv 2020:1–29.

18. Stoddard M, Sarkar S, Yuan L, Nolan RP, White DE, White LF, et al. Beyond the new normal: Assessing the feasibility of vaccine-based suppression of SARS-CoV-2. PLoS One 2021;16:e0254734. doi:10.1371/journal.pone.0254734.

19. Pinto Neto O, Kennedy DM, Reis JC, Wang Y, Brizzi ACB, Zambrano GJ, et al. Mathematical model of COVID-19 intervention scenarios for São Paulo—Brazil. Nat Commun 2021;12:1–13. doi:10.1038/s41467-020-20687-y.

20. Lyra W, José-Dias do Nascimento J, Belkhiria J, Almeida L de, Chrispim PPM, Andrade I de. COVID-19 pandemics modeling with SEIR(+CAQH), social distancing, and age stratification. The effect of vertical confinement and release in Brazil. MedRxiv 2020:2020.04.09.20060053. doi:10.1101/2020.04.09.20060053.

21. Hyun Mo Yang, Luis Pedro Lombardi Junior ACY. Are the SIR and SEIR models suitable to estimate the basic reproduction number for the CoViD-19 epidemic? MedRxiv 2020:2020.10.11.20210831.

22. Nash B, Badea A, Reddy A, Bosch M, Salcedo N, Gomez AR, et al. Validating and modeling the impact of high-frequency rapid antigen screening on COVID-19 spread and outcomes. MedRxiv 2021:2020.09.01.20184713. doi:10.1101/2020.09.01.20184713.

23. Oliveira JF, Jorge DCP, Veiga R V, Rodrigues MS, Torquato MF, da Silva NB, et al. Evaluating the burden of COVID-19 on hospital resources in Bahia, Brazil: A modelling-based analysis of 148 million individuals (preprint). MedRxiv 2020;2:2020.05.25.20105213.

24. Amaku M, Covas DT, Coutinho FAB, Azevedo RS, Massad E. Modelling the impact of contact tracing of symptomatic individuals on the COVID-19 epidemic. Clinics (Sao Paulo) 2021;76:e2639. doi:10.6061/clinics/2021/e2639.

25. Libotte GB, dos Anjos L, Almeida RCC, Malta SMC, Silva RS. Framework for enhancing the estimation of model parameters for data with a high level of uncertainty. Nonlinear Dyn 2022;107:1919–36. doi:10.1007/s11071-021-07069-9.

26. Pazos F, E. Felicioni F. A Control Approach to the Covid-19 Disease Using a SEIHRD Dynamical Model 2020:1–23. doi:10.48011/asba.v2i1.1002.

27. Crokidakis N. COVID-19 spreading in Rio de Janeiro, Brazil: Do the policies of social isolation really work? Chaos, Solitons and Fractals 2020;136. doi:10.1016/j.chaos.2020.109930.

28. Pacheco PMCL, Savi MA, Savi PV. Coronavirus disease 2019 (COVID-19) dynamics considering the influence of hospital infrastructure. MedRxiv 2020;2019:1–28. doi:10.1101/2020.06.03.20121608.

29. Thompson J, Wattam S. Estimating the impact of interventions against COVID-19: From lockdown to vaccination. PLoS One 2021;16:1–50. doi:10.1371/journal.pone.0261330.

30. Dias S, Queiroz K, Araujo A. Controlling epidemic diseases based only on social distancing level: General case 2020.

31. de Souza Melo A, da Penha Sobral AIG, Marinho MLM, Duarte GB, Vieira AA, Sobral MFF. The impact of social distancing on COVID-19 infections and deaths. Trop Dis Travel Med Vaccines 2021;7:1–7. doi:10.1186/s40794-021-00137-3.

32. Moghadas SM, Vilches TN, Zhang K, Nourbakhsh S, Sah P, Fitzpatrick MC, et al. Evaluation of COVID-19 vaccination strategies with a delayed second dose. PLoS Biol 2021;19:1–13. doi:10.1371/journal.pbio.3001211.

33. Nuno R. Faria, Thomas A. Mellan, Charles Whittaker IMC, Darlan da S. Candido, Swapnil Mishra, Myuki A. E. Crispim FCSS, Iwona Hawryluk, John T. McCrone, Ruben J. G. Hulswit LAMF, Mariana S. Ramundo, Jaqueline G. de Jesus, Pamela S. Andrade TMC, Giulia M. Ferreira, Camila A. M. Silva, Erika R. Manuli, Rafael H. M. Pereira, Pedro S. Peixoto, Moritz U. G. Kraemer, Nelson Gaburo Jr., Cecilia da C. Camilo, Henrique Hoeltgebaum, William M. Souza, Esmenia C. Rocha, Leandro M. de Souza, Mariana C. de Pi J do PS, Danielle A. G. Zauli, Alessandro C. de S. Ferreira, Ricardo P. Schnekenberg, Daniel J. Laydon, Patrick G. T. Walker, Hannah M. Schlüter, Ana L. P. dos Santos MSV, et al. Genomics and epidemiology of the P.1 SARS-CoV-2 lineage in Manaus, Brazil. Science (80- ) 2021;372:815–21.

34. Braga M de B, Fernandes R da S, Souza GN de, Rocha JEC da, Dolácio CJF, Tavares I da S, et al. Artificial neural networks for short-term forecasting of cases, deaths, and hospital beds occupancy in the COVID-19 pandemic at the Brazilian Amazon. PLoS One 2021;16:e0248161. doi:10.1371/journal.pone.0248161.

35. Dos Santos Gomes DC, De Oliveira Serra GL. Machine Learning Model for Computational Tracking and Forecasting the COVID-19 Dynamic Propagation. IEEE J Biomed Heal Informatics 2021;25:615–22. doi:10.1109/JBHI.2021.3052134.

36. Efimov D, Ushirobira R. On an interval prediction of COVID-19 development based on a SEIR epidemic model. Annu Rev Control 2021.

37. Aronna MS, Guglielmi R, Moschen LM. A model for COVID-19 with isolation, quarantine and testing as control measures. Epidemics 2021;34:100437. doi:10.1016/j.epidem.2021.100437.

38. Borghi PH, Zakordonets O, Teixeira JP, Henrique P, Zakordonets O, Paulo J. A COVID-19 time series forecasting model based on MLP ANN. Procedia Comput Sci 2020.

39. Gondim JAM. Preventing epidemics by wearing masks: An application to COVID-19. Chaos, Solitons and Fractals 2021.

40. Mac S, Mishra S, Ximenes R, Barrett K, Khan YA, Naimark DMJ, et al. Modeling the coronavirus disease 2019 pandemic: A comprehensive guide of infectious disease and decision-analytic models. J Clin Epidemiol 2021;132:133–41. doi:10.1016/j.jclinepi.2020.12.002.

41. Aguas R, White L, Hupert N, Shretta R, Pan-Ngum W, Celhay O, et al. Modelling the COVID-19 pandemic in context: an international participatory approach. BMJ Glob Heal 2020;5. doi:10.1136/bmjgh-2020-003126.

42. Friston K, Costello A, Pillay D. “Dark matter”, second waves and epidemiological modelling. BMJ Glob Heal 2020;5:1–13. doi:10.1136/bmjgh-2020-003978.

43. Andrade LA, Gomes DS, Lima SVMA, Duque AM, Melo MS, Góes MAO, et al. COVID-19 Mortality in an area of northeast brazil: Epidemiological characteristics and prospective spatiotemporal modeling. Epidemiol Infect 2020. doi:10.1017/S0950268820002915.

44. Kayral İE, Buzrul S. Forecasting of COVID-19 infections in E7 countries and proposing some policies based on the stringency index. J Popul Ther Clin Pharmacol 2020;27:e76–84. doi:10.15586/JPTCP.V27ISP1.757.

45. Gomes DS, Andrade LA, Ribeiro CJN, Peixoto MVS, Lima SVMA, Duque AM, et al. Risk clusters of COVID-19 transmission in Northeastern Brazil: Prospective space-time modeling. Epidemiol Infect 2020. doi:10.1017/S0950268820001843.

46. Hazarika BB, Gupta D. Modelling and forecasting of COVID-19 spread using wavelet-coupled random vector functional link networks. Appl Soft Comput J 2020.

47. Sahai KA, Rath N, Sood V, Pratap M. ARIMA modelling & forecasting of COVID-19 in top five affected countries 2020.

48. Yang HM, Lombardi Junior LP, Castro FFM, Yang AC. Mathematical model describing CoViD-19 in São Paulo State, Brazil - Evaluating isolation as control mechanism and forecasting epidemiological scenarios of release. Epidemiol Infect 2020. doi:10.1017/S0950268820001600.

49. Wang P, Zheng X, Li J, Zhu B. Prediction of epidemic trends in COVID-19 with logistic model and machine learning technics. Chaos, Solitons and Fractals 2020;139:110058. doi:10.1016/j.chaos.2020.110058.

50. Starling C, Couto B, JúniorJJ C, Alvim A, Oliveira C, Souza L, et al. Mobility restrictions and COVID-19 pandemic outbreak control 2020;7:625–6.

51. Ankaralı H, Erarslan N, Pasin Ö, Al-Mahmood AK. Modeling and short-term forecasts of indicators for COVID-19 outbreak in 25 countries at the end of march. Bangladesh J Med Sci 2020;19:6–20. doi:10.3329/bjms.v19i0.47611.

52. Jorge DCP, Rodrigues MS, Silva MS, Cardim LL, da Silva NB, Silveira IH, et al. Assessing the nationwide impact of COVID-19 mitigation policies on the transmission rate of SARS-CoV-2 in Brazil. Epidemics 2021;35:100465. doi:10.1016/j.epidem.2021.100465.

53. Gonzalez-Parra G, Martínez-Rodríguez D, Villanueva-Micó R. Impact of a New SARS-CoV-2 Variant on the Population: A Mathematical Modeling Approach. Math Comput Appl 2021;26:25. doi:10.3390/mca26020025.

54. Pereira IG, Guerin JM, Júnior AGS, Garcia GS, Piscitelli P, Miani A, et al. Forecasting covid-19 dynamics in brazil: A data driven approach. Int J Environ Res Public Health 2020;17:1–26. doi:10.3390/ijerph17145115.

55. de Oliveira ACS, Morita LHM, da Silva EB, Zardo LAR, Fontes CJF, Granzotto DCT. Bayesian modeling of COVID-19 cases with a correction to account for under-reported cases. Infect Dis Model 2020;5:699–713. doi:10.1016/j.idm.2020.09.005.

56. Batistela CM, Correa DPF, Bueno ÁM, Piqueira JRC. SIRSi-Vaccine dynamical model for Covid-19 pandemic 2021.

57. Batista AA, da Silva SH. An Epidemiological Compartmental Model With Automated Parameter Estimation and Forecasting of the Spread of COVID-19 With Analysis of Data From Germany and Brazil. Front Appl Math Stat 2022;8:1–37. doi:10.3389/fams.2022.645614.

58. Rodrigues T, Helene O. Monte Carlo approach to model COVID-19 deaths and infections using Gompertz functions. Phys Rev Res 2020;2:1–8. doi:10.1103/PhysRevResearch.2.043381.

59. LAZO MJ, CEZARO ADE. Why can we observe a plateau even in an out of control epidemic outbreak? A SEIR model with the interaction of *n* distinct populations for COVID-19 in Brazil. Trends Comput Appl Math 2021;22.

60. Morato MM, Pataro IML, Da Costa M, Normey-Rico JE. Optimal Control Concerns Regarding the COVID-19 (SARS-CoV-2) Pandemic in Bahia and Santa Catarina, Brazil. ArXiv Prepr ArXiv 2020. doi:https://doi.org/10.48550/arXiv.2006.14108.

61. Gonzales RE. Different scenarios in the Dynamics of SARS-Cov-2 Infection: an adapted ODE model. ArXiv Prepr ArXiv 2020. doi:https://doi.org/10.48550/arXiv.2004.01295.

**Legend:** Electronic and additional searches retrieved 1061 references. After removing duplicates (127), titles and abstracts of 934 references were screened, leading to a selection of (156) full texts. Of those (156) studies that were assessed for eligibility, seventy-five (75) were excluded for various reasons, comprising finally 81 studies included in this review. The list of excluded studies at the full-text reading stage and the reasons for exclusion are presented in S3 Table.
