# Supplementary material for: Dynamic transmission modeling of COVID-19 to support decision-making in Brazil: A scoping review in the pre-vaccine era
Source: PLOS Glob Public Health. 2023 Dec 13;3(12):e0002679. doi: 10.1371/journal.pgph.0002679 (PMC10718415; doi:10.1371/journal.pgph.0002679)
Supplement: S4 Table — Considering that parameters directly influence the performance of the models and the obtained outputs, an assessment of the parameter sources was conducted. Parameters were categorized as follows: extracted from literary sources, obtained from government and non-governmental data sources, and directly used in the model without any new adjustments (assumed); calculated and adjusted from secondary data sources (estimated from secondary data); or estimated from the evaluated model itself (modeled). The main parameters evaluated, their sources, and the references used are listed in Table 4 (main text). All data sources used by the evaluated articles were assessed and are listed in S4 Table. (DOCX) [file pgph.0002679.s004.docx]

**Parameters’ Data Sources**

**Table S4: Data sources assessed for the models’ parameters.**

| **Parameters** | **Main source of data for estimated and assumed parameters** | **References** |
| --- | --- | --- |
| **R0/Re** | Brazilian Ministry of Health; Brasil.io;  São Paulo government; Johns Hopkins University; Scientific literature. | [1-13] |
| **Serial Interval** | Scientific literature. | [14] |
| **Incubation Period** | WHO; CDC; Scientific literature. | [12,15–33] |
| **Infectious Period** | Brazilian Ministry of Health; WHO; CDC; Scientific literature. | [17,34–45] |
| **Case fatality rate** | Worldometer; Johns Hopkins University; WHO; Brazilian Ministry of Health; ANVISA; Centro de coordinación de alertas y emergencias sanitarias - Gobierno Españaa; SEADE - São Paulo Government; Distrito Federal Government; Scientific literature. | [37,46-53] |
| **Proportion of suceptible** | Worldometer; Johns Hopkins University; WHO; SIVEP- SRAG; Brasil.io; Brazilian Ministry of Health; IBGE; SEADE - São Paulo Government; Scientific literature. | [54–58] |
| **Mobility** | SEADE - São Paulo Government; OAG airline data; Inloco technology company; Google Community Mobility; Apple Maps. | [59-62] |
| **Contact rate** | Brazilian Ministry of Health; SEADE - São Paulo Government; Scientific literature. | [22,63–66] |
| **Birth/death rate** | SEADE - São Paulo Government; IBGE. | [67][68] |
| **Healthcare capacity** | AMIB; Brazilian Ministry of Health; SEADE - São Paulo Government; SESAB; WHO; OECD; DATASUS. | [69–75] |

**References**

1. Brazilian Ministry of Health. Coronavirus panel. 2020.

2. Ministério da saúde-Brasil. Boletim Epidemiológico 08 2020. https://www.saude.gov.br/images/pdf/2020/April/09/be-covid-08-final-2.pdf.

3. de Souza WM, Buss LF, Candido D da S, Carrera J-P, Li S, Zarebski AE, et al. Epidemiological and clinical characteristics of the COVID-19 epidemic in Brazil. Nat Hum Behav 2020;4:856–65. doi:10.1038/s41562-020-0928-4.

4. Yang HM, Junior LPL, Castro FFM, Yang AC. ̃Evaluating reduction in CoViD-19 cases by isolation and protective measures in São Paulo State, Brazil, and scenarios of release. MedRxiv 2020:2020.05.19.20099309. doi:10.1101/2020.05.19.20099309.

5. Liu Y, Gayle AA, Wilder-Smith A, Rocklöv J. The reproductive number of COVID-19 is higher compared to SARS coronavirus. J Travel Med 2020;27. doi:10.1093/jtm/taaa021.

6. Brasil.io. Open data in Brazil 2020. https://brasil.io/home/.

7. Cidade de São Paulo Saúde. Boletins COVID-19. São Paulo, Brazi 2020. https://www.prefeitura.sp.gov.br/cidade/secretarias/ saude/vigilancia_em_saude/doencas_e_agravos/coronavirus/index. php?p=295572.

8. Saúde SP (Cidade). S da. Casos em Sao Paulo; Database. 2020. https://www.saopaulo.sp.gov.br/coronavirus#numero-vacinacao.

9. SEADE. SEADE, SP contra o novo coronavırus – Boletim completo 2020. https://www.seade.gov.br/coronavirus/?utm source=portal&utm medium=banner&utm campaign=boletim-completo.

10. Johns Hopkins University. COVID-19 Dashboard by the Center for Systems Science and Engineering (CSSE) at Johns Hopkins University (JHU). 2020 n.d.

11. Wu JT, Leung K, Leung GM. Nowcasting and forecasting the potential domestic and international spread of the 2019-nCoV outbreak originating in Wuhan, China: a modelling study. Lancet (London, England) 2020;395:689–97. doi:10.1016/S0140-6736(20)30260-9.

12. Li Q, Guan X, Wu P, Wang X, Zhou L, Tong Y, et al. Early Transmission Dynamics in Wuhan, China, of Novel Coronavirus–Infected Pneumonia. N Engl J Med 2020;382:1199–207. doi:10.1056/nejmoa2001316.

13. Abbott S, Hellewell J, Munday J, Funk S. The transmissibility of novel Coronavirus in the early stages of the 2019-20 outbreak in Wuhan: Exploring initial point-source exposure sizes and durations using scenario analysis. Wellcome Open Res 2020;5:17. doi:10.12688/wellcomeopenres.15718.1.

14. Nishiura H, Linton NM, Akhmetzhanov AR. Serial interval of novel coronavirus (COVID-19) infections. Int J Infect Dis 2020;93:284–6. doi:https://doi.org/10.1016/j.ijid.2020.02.060.

15. World Health Organization (WHO). Report of the WHO-China Joint Mission on Coronavirus Disease 2019 (COVID-19),16–24 February 2020 2020.

16. Boldog P, Tekeli T, Vizi Z, Dénes A, Bartha FA, Röst G. Risk Assessment of Novel Coronavirus COVID-19 Outbreaks Outside China. J Clin Med 2020;9. doi:10.3390/jcm9020571.

17. Lauer SA, Grantz KH, Bi Q, Jones FK, Zheng Q, Meredith HR, et al. The Incubation Period of Coronavirus Disease 2019 (COVID-19) From Publicly Reported Confirmed Cases: Estimation and Application. Ann Intern Med 2020;172:577–82. doi:10.7326/M20-0504.

18. Liu Z, Magal P, Webb G. Predicting the number of reported and unreported cases for the COVID-19 epidemics in China, South Korea, Italy, France, Germany and United Kingdom. J Theor Biol 2021;509:110501. doi:10.1016/j.jtbi.2020.110501.

19. Kucharski AJ, Russell TW, Diamond C, Liu Y, Edmunds J, Funk S, et al. Early dynamics of transmission and control of COVID-19: a mathematical modelling study. Lancet Infect Dis 2020;20:553–8. doi:10.1016/S1473-3099(20)30144-4.

20. Ferguson N, Laydon D, Nedjati Gilani G, Imai N, Ainslie K, Baguelin M, et al. Report 9: Impact of non-pharmaceutical interventions (NPIs) to reduce COVID19 mortality and healthcare demand. 2020. doi:10.25561/77482.

21. Wei Y, Wei L, Liu Y, Huang L, Shen S, Zhang R, et al. A systematic review and meta-analysis reveals long and dispersive incubation period of COVID-19 2020. doi:10.1101/2020.06.20.20134387.

22. Castilho C, Gondim JAM, Marchesin M, Sabeti M. Assessing the efficiency of different control strategies for the coronavirus (COVID-19) epidemic. ArXiv Prepr ArXiv200403539 2020.

23. Kissler SM, Tedijanto C, Goldstein E, Grad YH, Lipsitch M. Projecting the transmission dynamics of SARS-CoV-2 through the postpandemic period. Science 2020;368:860–8. doi:10.1126/science.abb5793.

24. Sanche S, Lin YT, Xu C, Romero-Severson E, Hengartner N, Ke R. High Contagiousness and Rapid Spread of Severe Acute Respiratory Syndrome Coronavirus 2. Emerg Infect Dis J 2020;26:1470. doi:10.3201/eid2607.200282.

25. Li R, Pei S, Chen B, Song Y, Zhang T, Yang W, et al. Substantial undocumented infection facilitates the rapid dissemination of novel coronavirus (SARS-CoV-2). Science 2020;368:489–93. doi:10.1126/science.abb3221.

26. Centers for Disease Control and Prevention (CDC). Coronavirus Disease 2019 (COVID-2019). 2020. https://www.cdc.gov/coronavirus/2019-ncov/symptoms-testing/symptoms.html.

27. Ferretti L, Wymant C, Kendall M, Zhao L, Nurtay A, Abeler-Dörner L, et al. Quantifying SARS-CoV-2 transmission suggests epidemic control with digital contact tracing. Science 2020;368. doi:10.1126/science.abb6936.

28. Linton NM, Kobayashi T, Yang Y, Hayashi K, Akhmetzhanov AR, Jung S-M, et al. Incubation Period and Other Epidemiological Characteristics of 2019 Novel Coronavirus Infections with Right Truncation: A Statistical Analysis of Publicly Available Case Data. J Clin Med 2020;9. doi:10.3390/jcm9020538.

29. Spencer JA, Shutt DP, Moser SK, Clegg H, Wearing HJ, Mukundan H, et al. Epidemiological parameter review and comparative dynamics of influenza, respiratory syncytial virus, rhinovirus, human coronavirus, and adenovirus. MedRxiv 2020:2020.02.04.20020404. doi:10.1101/2020.02.04.20020404.

30. Lin Q, Zhao S, Gao D, Lou Y, Yang S, Musa SS, et al. A conceptual model for the coronavirus disease 2019 (COVID-19) outbreak in Wuhan, China with individual reaction and governmental action. Int J Infect Dis IJID Off Publ Int Soc Infect Dis 2020;93:211–6. doi:10.1016/j.ijid.2020.02.058.

31. Hao X, Cheng S, Wu D, Wu T, Lin X, Wang C. Reconstruction of the full transmission dynamics of COVID-19 in Wuhan. Nature 2020;584:420–4. doi:10.1038/s41586-020-2554-8.

32. Backer JA, Klinkenberg D, Wallinga J. Incubation period of 2019 novel coronavirus (2019-nCoV) infections among travellers from Wuhan, China, 20-28 January 2020. Euro Surveill 2020;25. doi:10.2807/1560-7917.es.2020.25.5.2000062.

33. Guan W, Ni Z, Hu Y, Liang W, Ou C, He J, et al. Clinical Characteristics of Coronavirus Disease 2019 in China. N Engl J Med 2020;382:1708–20. doi:10.1056/NEJMoa2002032.

34. World Health Organization (WHO). Novel coronavirus (2019-nCoV). 2020. https://www.who.int/docs/default- source/ coronaviruse/situation-reports/20200315-sitrep-55-COVID-19.pdfsfvrsn= 33daa5cb_8.

35. Zhou F, Yu T, Du R, Fan G, Liu Y, Liu Z, et al. Clinical course and risk factors for mortality of adult inpatients with COVID-19 in Wuhan, China: a retrospective cohort study. Lancet (London, England) 2020;395:1054–62. doi:10.1016/S0140-6736(20)30566-3.

36. Cevik M, Tate M, Lloyd O, Maraolo AE, Schafers J, Ho A. SARS-CoV-2, SARS-CoV-1 and MERS-CoV viral load dynamics, duration of viral shedding and infectiousness: a living systematic review and meta-analysis. MedRxiv 2020:2020.07.25.20162107. doi:10.1101/2020.07.25.20162107.

37. Verity R, Okell LC, Dorigatti I, Winskill P, Whittaker C, Imai N, et al. Estimates of the severity of coronavirus disease 2019: a model-based analysis. Lancet Infect Dis 2020;20:669–77. doi:10.1016/S1473-3099(20)30243-7.

38. Byrne AW, McEvoy D, Collins AB, Hunt K, Casey M, Barber A, et al. Inferred duration of infectious period of SARS-CoV-2: rapid scoping review and analysis of available evidence for asymptomatic and symptomatic COVID-19 cases. BMJ Open 2020;10:e039856. doi:10.1136/bmjopen-2020-039856.

39. Liu T, Hu J, Kang M, Lin L, Zhong H, Xiao J, et al. Transmission dynamics of 2019 novel coronavirus (2019-nCoV). BioRxiv 2020:2020.01.25.919787. doi:10.1101/2020.01.25.919787.

40. Yang X, Yu Y, Xu J, Shu H, Xia J, Liu H, et al. Clinical course and outcomes of critically ill patients with SARS-CoV-2 pneumonia in Wuhan, China: a single-centered, retrospective, observational study. 2020:U2-.

41. Read JM, Bridgen JRE, Cummings DAT, Ho A, Jewell CP. Novel coronavirus 2019-nCoV (COVID-19): early estimation of epidemiological parameters and epidemic size estimates. Philos Trans R Soc London Ser B, Biol Sci 2021;376:20200265. doi:10.1098/rstb.2020.0265.

42. Arons MM, Hatfield KM, Reddy SC, Kimball A, James A, Jacobs JR, et al. Presymptomatic SARS-CoV-2 Infections and Transmission in a Skilled Nursing Facility. N Engl J Med 2020;382:2081–90. doi:10.1056/NEJMoa2008457.

43. Acuña-Zegarra MA, Santana-Cibrian M, Velasco-Hernandez JX. Modeling behavioral change and COVID-19 containment in Mexico: A trade-off between lockdown and compliance. Math Biosci 2020;325:108370. doi:10.1016/j.mbs.2020.108370.

44. Bhatraju PK, Ghassemieh BJ, Nichols M, Kim R, Jerome KR, Nalla AK, et al. Covid-19 in Critically Ill Patients in the Seattle Region - Case Series. N Engl J Med 2020;382:2012–22. doi:10.1056/NEJMoa2004500.

45. Tang B, Wang X, Li Q, Bragazzi NL, Tang S, Xiao Y, et al. Estimation of the Transmission Risk of the 2019-nCoV and Its Implication for Public Health Interventions. J Clin Med 2020;9. doi:10.3390/jcm9020462.

46. Worldometer. COVID-19 Coronavirus Pandemic 2020. https://www.worldometers.info/coronavirus/ .

47. São Paulo. Secretária Estadual de Saúde. Sistema de Monitoramento Inteligente do Estado de Sao Paulo 2020. https://www.simi.sp.gov.br/#/views/PainelCovid-19/MunicpiosDetalhado?:iid1⁄41.

48. Distrito Federal. Secretária de Saúde. Boletins Informativos DIVEP/CIEVES (COE). Boletins informativos sobre coronavirus (COVID-19). 2020.

49. Anvisa. M da S-MAN de VS. Coronavírus Brasil 2020. https://covid.saude.gov.br/ .

50. Novel Coronavirus Pneumonia Emergency Response Epidemiology Team. Zhonghua Liu Xing Bing Xue Za Zhi. The Epidemiological Characteristics of an Outbreak of 2019 Novel Coronavirus Diseases (COVID-19) in China. 2020;41:45–151. doi:10.3760/cma.j.issn.0254.

51. Silva AAM da, Lima-Neto LG, Azevedo C de MPES de, Costa LMM da, Bragança MLBM, Barros Filho AKD, et al. Population-based seroprevalence of SARS-CoV-2 and the herd immunity threshold in Maranhão. Rev Saude Publica 2020;54:131. doi:10.11606/s1518-8787.2020054003278.

52. Centro de coordinacion de alertas y emergencias sanitarias goberno espana. Enfermedad por el coronavirus (covid-19). 2020.

53. Salje H, Tran Kiem C, Lefrancq N, Courtejoie N, Bosetti P, Paireau J, et al. Estimating the burden of SARS-CoV-2 in France. Science 2020;369:208–11. doi:10.1126/science.abc3517.

54. Instituto Brasileiro de Geografia e Estatística. (IBGE). Estimativas populacionais n.d. https://www.ibge.gov.br/estatisticas/sociais/populacao/9103- estimativas-de-populacao.html?=&t=downloads.

55. BRASIL. Ministério da Saúde. Banco de Dados de Síndrome Respiratória Aguda Grave - incluindo dados da COVID-19 n.d. https://opendatasus.saude.gov.br/ (accessed December 10, 2020).

56. Ritchie H. Our world in data. 2020.

57. Lalwani P, Salgado BB, Filho IVP, da Silva DSS, de Morais TB do N, Jordão MF, et al. SARS-CoV-2 seroprevalence and associated factors in Manaus, Brazil: baseline results from the DETECTCoV-19 cohort study. Int J Infect Dis IJID Off Publ Int Soc Infect Dis 2021;110:141–50. doi:10.1016/j.ijid.2021.07.017.

58. Bastos SB, Morato MM, Cajueiro DO, Normey-Rico JE. The COVID-19 (SARS-CoV-2) Uncertainty Tripod in Brazil: Assessments on model-based predictions with large under-reporting. Alexandria Eng J 2021;60:4363–80.

59. InLoco. Mapa brasileiro da Covid-19 2020. https://mapabrasileirodacovid.inloco.com.br/pt.

60. Apple. Mobility Trends Reports 2020.

61. OAG. Official Airline Guide. 2020. http://www.oag.com.

62. Google. COVID-19. Community Mobility Report. 2020.

63. Arregui S, Aleta A, Sanz J, Moreno Y. Projecting social contact matrices to different demographic structures. PLoS Comput Biol 2018;14:e1006638. doi:10.1371/journal.pcbi.1006638.

64. Mossong J, Hens N, Jit M, Beutels P, Auranen K, Mikolajczyk R, et al. Social contacts and mixing patterns relevant to the spread of infectious diseases. PLoS Med 2008;5:e74. doi:10.1371/journal.pmed.0050074.

65. Yang HM. Directly transmitted infections modeling considering an age-structured contact rate. Math Comput Model 1999;29:39–48. doi:https://doi.org/10.1016/S0895-7177(99)00069-2.

66. Prem K, Cook AR, Jit M. Projecting social contact matrices in 152 countries using contact surveys and demographic data. PLoS Comput Biol 2017;13:e1005697. doi:10.1371/journal.pcbi.1005697.

67. Instituto Brasileiro de Geografia e Estatística (IBGE). Tábuas completas de mortalidade 2020.

68. Instituto Brasileiro de Geografia e Estatística (IBGE). Sistema IBGE de Recuperação Automática - SIDRA. Estatísticas do registro civil. n.d. https://sidra.ibge.gov.br/tabela/2679 (accessed November 12, 2020).

69. AMIB. Associação de Medicina Intensiva Brasileira 2018. https://www.amib.org.br/.

70. Ministério da Saúde (Brasil). Secretaria de Atenção à Saúde. CNESnet. Consulta a leitos n.d. http://cnes2.datasus.gov.br/Mod_Ind_Tipo_Leito.

71. State Secretary of Health of Bahia (SESAB). Covid-19 n.d. https://infovis.sei.ba.gov.br/covid19/ .

72. OECD. Hospital beds(indicator) 2020. https://doi.org/10.1787/0191328e-en.

73. Brazilian Federal Medicine Council. ICU beds in Brazil 2020.

74. de Carvalho BS, Silva LFC, Matarazzo H, Zoca H, Melo H. CENÁRIO DOS HOSPITAIS NO BRASIL. Fed Bras Hosp 2019.

75. Ministério da Saúde (Brasil). Cadastro Nacional do Estabelecimentos de Saúde-DATASUS 2019. ftp://ftp.datasus.gov.br/dissemin/publicos/cnes/200508_/dados/LT.

**Legend:** Considering that parameters directly influence the performance of the models and the obtained outputs, an assessment of the parameter sources was conducted. Parameters were categorized as follows: extracted from literary sources, obtained from government and non-governmental data sources, and directly used in the model without any new adjustments (assumed); calculated and adjusted from secondary data sources (estimated from secondary data); or estimated from the evaluated model itself (modeled). The main parameters evaluated, their sources, and the references used are listed in Table 4 (main text). All data sources used by the evaluated articles were assessed and are listed in S4 Table.
