# Supplementary material for: Dynamic transmission modeling of COVID-19 to support decision-making in Brazil: A scoping review in the pre-vaccine era
Source: PLOS Glob Public Health. 2023 Dec 13;3(12):e0002679. doi: 10.1371/journal.pgph.0002679 (PMC10718415; doi:10.1371/journal.pgph.0002679)
Supplement: S5 Table — As calibration plays a pivotal role in the modeling process, particularly with the level of reliability required for COVID-19 decision-making, approximately 70% of the articles provided details of their calibration parameters and the data employed in the process. Notably, about 30% of the reviewed articles omitted any reference to their fitting step. Among the articles that did discuss model calibration, we compiled in S5 Table the key parameters that were adjusted, as well as a comprehensive list of the primary data sources used for calibration. (DOCX) [file pgph.0002679.s005.docx]

**Calibration**

**S5 Table: Reporting of calibration parameters and primary data sources**

| Main calibrated parameters | Proportion of articles | List of main data sources used | | |  |
| --- | --- | --- | --- | --- | --- |
|  |  |  |  |  |  |
| Transmission rate | 23% | <https://brasil.io/home/(2020)> | | |  |
| Mortality rate | 12% | [https://datasus.saude.gov.br](https://datasus.saude.gov.br/) |  |  |  |
| Number of infections | 26% | <https://www.worldometers.info/> | | |  |
| Recovery rate | 19% | <https://www.who.int/countries/bra/> | | |  |
| Hospitalization | 11% | [https://www.seade.gov.br](https://www.seade.gov.br/) | | |  |
| Case-fatality rate | 6% | <https://infovis.sei.ba.gov.br/covid19/> | | |  |
| Age structure | 6% | <https://data.oecd.org/healtheqt/hospital-beds.htm> | | |  |
| Infectious contacts | 13% | <https://coronavirus.jhu.edu/map.html> | | |  |
| Number of deaths | 13% | <https://www.saopaulo.sp.gov.br/planosp/simi/> | | |  |
| Social distancing | 11% | [https://covid.saude.gov.br](https://covid.saude.gov.br/) | | |  |
| Incubation period | 4% |  | | |  |

**Legend:** As calibration plays a pivotal role in the modeling process, particularly with the level of reliability required for COVID-19 decision-making, approximately 70% of the articles provided details of their calibration parameters and the data employed in the process. Notably, about 30% of the reviewed articles omitted any reference to their fitting step. Among the articles that did discuss model calibration, we compiled in Table S5 the key parameters that were adjusted, as well as a comprehensive list of the primary data sources used for calibration.
