# Supplementary material for: Dynamic transmission modeling of COVID-19 to support decision-making in Brazil: A scoping review in the pre-vaccine era
Source: PLOS Glob Public Health. 2023 Dec 13;3(12):e0002679. doi: 10.1371/journal.pgph.0002679 (PMC10718415; doi:10.1371/journal.pgph.0002679)
Supplement: S1 Table — To be included in this review, studies needed to present COVID-19 or SARS-CoV-2 modeling in Brazil, including infectious disease modeling methods. Peer-reviewed journals papers, gray literature, or database repositories files were included if they were: published between January 01, 2020, and June 2, 2021, written in any language, involved COVID-19 analyses and modeling methodology (including analytical decision models, cohort and Markov models, compartmental models, and individual agent-based modeling), reporting real-world or scenario-based COVID-19 modeling, for Brazil. Papers were excluded if they did not fit the study’s conceptual framework (modeling of non-COVID outcomes) or were not fully available (works not fully retrieved or poster abstracts). Case reports, case series, guidelines/recommendations, letters/perspective/editorials/comments pieces, reviews, and descriptive epidemiological publications were excluded. Studies presenting only mathematical models or software tools but not modeling outputs, projections, results, or works using projections only as examples of the models’ application were excluded. Studies modeling vaccine outputs were also excluded. (DOCX) [file pgph.0002679.s001.docx]

**Inclusion and exclusion criteria**

**S1 Table:** **Inclusion and exclusion criteria.**

| **Inclusion criteria** | **Exclusion criteria** |
| --- | --- |
| All data derived from or studies reporting on COVID or SARS-CoV-2 modeling, including infectious disease modeling methods, mathematical modeling methods including analytical decision models, cohort and Markov models, compartmental and individual agent-based modeling, reporting real-world or scenario-based COVID-19 models in Brazil. | Modeling of non-COVID-19 outcomes.  Modeling of COVID-19 vaccine outcomes.  Studies which only present mathematical and/or statistical models or software tools, but not modeling outputs, projections, or results. |
| Model outputs which include transmission and epidemic trajectory of SARS-CoV-2 and/or COVID-19 reported as any relevant epidemiological output during the COVID-19 pandemic in 2020 and 2021, in Brazil. These may include but are not limited to SARS-CoV-2 infections (number and rates), confirmed COVID-19 cases, cases and deaths due to SARS, hospitalization due to COVID-19, and deaths due to COVID-19, as well as healthcare service demand including hospital beds (conventional and ICU), ventilators and others. | Descriptive epidemiological publications (e.g. describing case positive proportions, total case numbers, descriptive mapping of incidence by geographic information systems), not derived from modeling. |
| Published and non-published studies, data presented in repository and databases, and technical reports. No restriction of location or language were applied in the literature search. | Data repositories, technical reports or studies with abstract or full text not available. Case reports, case series, guidelines and recommendations, letters, perspectives, editorials, comments pieces, and reviews were excluded. |

**Legend:** To be included in this review, studies needed to present COVID-19 or SARS-CoV-2 modeling in Brazil, including infectious disease modeling methods. Peer-reviewed journals papers, gray literature, or database repositories files were included if they were: published between January 01, 2020, and June 2, 2021, written in any language, involved COVID-19 analyses and modeling methodology (including analytical decision models, cohort and Markov models, compartmental models, and individual agent-based modeling), reporting real-world or scenario-based COVID-19 modeling, for Brazil. Papers were excluded if they did not fit the study's conceptual framework (modeling of non-COVID outcomes) or were not fully available (works not fully retrieved or poster abstracts). Case reports, case series, guidelines/recommendations, letters/perspective/editorials/comments pieces, reviews, and descriptive epidemiological publications were excluded. Studies presenting only mathematical models or software tools but not modeling outputs, projections, results, or works using projections only as examples of the models' application were excluded. Studies modeling vaccine outputs were also excluded.
