# Supplementary material for: Dynamic transmission modeling of COVID-19 to support decision-making in Brazil: A scoping review in the pre-vaccine era
Source: PLOS Glob Public Health. 2023 Dec 13;3(12):e0002679. doi: 10.1371/journal.pgph.0002679 (PMC10718415; doi:10.1371/journal.pgph.0002679)
Supplement: S2 Table — Search performed from May 31 to June 2, 2021. In order to identify the potentially relevant studies, search strategies were applied in MEDLINE (via PubMed) and adjusted for the other databases (Embase and LILACS). Gray literature (papers published but not peer-reviewed) was searched in ArXiv, Medrxiv, Biorxiv, and Open Gray. Repositories accessed were Figshare, Github, Zenodo, and Dryad. Additionally, reference lists of included studies were hand-searched to identify potentially eligible studies. The searches were carried out from May 31 to June 2, 2021. (DOCX) [file pgph.0002679.s002.docx]

**Information Sources (Search Strategy)**

**S2 Table: Search strategy for COVID-19 models in Brazil, in a pre-vaccine era. Search performed from May 31 to June 2, 2021.**

| **Database** | **Search Strategy** | **Results** |
| --- | --- | --- |
| **MEDLINE**  (via PubMed)  05/31/2021 | #1 "COVID-19"[Supplementary Concept] OR "severe acute respiratory syndrome coronavirus 2"[Supplementary Concept] OR "severe acute respiratory syndrome coronavirus 2”    #2 "COVID-19"[Mesh] OR "SARS-CoV-2"[Mesh]    #3 “COVID 19” OR (COVID-19 Virus Disease) OR (COVID-19 Virus Infection) OR (2019-nCoV Infection) OR (Coronavirus Disease-19) OR (2019 Novel Coronavirus Disease) OR (2019 Novel Coronavirus Infection) OR (2019-nCoV Disease) OR (SARS Coronavirus 2 Infection) OR (SARS-CoV-2 Infection) OR (COVID-19 Pandemic)    #4 "Decision Support Techniques"[Mesh] OR "Models, Theoretical"[Mesh] OR (Infectious disease model) OR (Mathematical Model) OR ( modeling; methods) OR (epidemiological models) OR (transmission model) OR (predictive model) OR (simulation models) OR (infectious disease dynamics modelling) OR (SEIR model)  #5 "brazil"[MeSH Terms] OR "brazil/statistics and numerical data"[MeSH Terms] OR "brazil"[MeSH Terms] OR "brazil"[All Fields] OR "brazil s"[All Fields] OR "brazils"[All Fields] OR "brazil"[MeSH Terms] OR brazil*  #6 (#1 OR #2 OR #3) AND #4 AND 5 | 559 |
| **LILACS**  05/31/2021 | #1 (mh:('Coronavirus Infections')) OR 'COVID 19' OR 'COVID-19 Virus Disease' OR 'COVID-19 Virus Infection' OR '2019-nCoV Infection' OR 'Coronavirus Disease-19' OR '2019 Novel Coronavirus Disease' OR 'SARS-CoV-2'  #2 'Models, Theoretical' OR 'transmission model' OR 'predictive model' OR 'simulation models' OR 'infectious disease dynamics modelling' OR 'SEIR model' OR 'Modeling, Decision' OR 'modeling'  #3 ( mh:("Systems Analysis")) OR ( mh:("Decision Support Techniques"))  #4 #1 AND (#2 OR #3) | 17 |
| **Embase**  05/31/2021 | 1# 'coronavirus disease 2019'/exp OR ‘2019 novel coronavirus disease' OR ‘2019 novel coronavirus epidemic' OR ‘2019 novel coronavirus infection' OR '2019-nCoV disease' OR '2019-nCoV infection' OR 'coronavirus disease 2' OR 'COVID 19' OR 'SARS coronavirus 2 infection' OR 'SARS coronavirus 2 pneumonia' OR 'SARS-CoV-2 disease' OR 'severe acute respiratory syndrome 2' OR 'Wuhan coronavirus disease' OR 'Wuhan coronavirus infection' OR 'COVID-19 Pandemic'  #2 'decision support system'/exp OR 'predictive model'/exp OR 'mathematical model'/exp  #3 'infectious disease model' OR (mathematical AND model) OR (modeling; AND methods) OR (epidemiological AND models) OR (transmission AND model) OR (predictive AND model) OR (simulation AND models) OR (infectious AND disease AND dynamics AND modelling) OR (seir AND model) OR 'Models, Theoretical' OR 'transmission model' OR 'predictive model' OR 'simulation models' OR 'infectious disease dynamics modelling' OR 'SEIR model' OR 'Modeling, Decision' OR 'modeling'  #4 #2 OR #3  #5 'brazil'/exp OR 'federative republic of brazil' OR 'united states of brazil' OR brazil  #6 #1 AND #4 AND #5 AND [embase]/lim NOT ([embase]/lim AND [medline]/lim) | 53 |
| **ArXiv**  06/02/2021 | "COVID 19" OR "SARS-CoV-2" AND "predictive model" OR "modeling" OR "dynamics modelling" AND Brazil | 60 |
| **MedrXiv**  06/02/2021 | For abstract or title COVID 19 OR "SARS-CoV-2" AND ("SEIR model" OR "predictive model" OR "modeling" OR "dynamics modelling") AND Brazil" | 215 |
| **BiorXiv**  06/02/2021 | COVID 19 OR "SARS-CoV-2" AND ("SEIR model" OR "predictive model" OR " 'modeling") AND Brazil" | 4 |
| **Open Gray**  06/02/2021 | COVID 19 OR "SARS-CoV-2" AND ("SEIR model" OR "predictive model" OR " 'modeling") | 0 |
| **Figshare**  06/02/2021 | COVID AND Brazil AND model | 55 |
| **Github**  06/02/2021 | COVID AND Brazil OR Brasil AND SIR **in:readme** | 82 |
| **Zenodo**  06/02/2021 | COVID AND Brazil AND model | 16 |
| **Dryad**  06/02/2021 | COVID AND Brazil AND model | 0 |
| **Total** |  | **1061** |

**Legend:** In order to identify the potentially relevant studies, search strategies were applied in MEDLINE (via PubMed) and adjusted for the other databases (Embase and LILACS). Gray literature (papers published but not peer-reviewed) was searched in ArXiv, Medrxiv, Biorxiv, and Open Gray. Repositories accessed were Figshare, Github, Zenodo, and Dryad. Additionally, reference lists of included studies were hand-searched to identify potentially eligible studies. The searches were carried out from May 31 to June 2, 2021.
